# Macro-Economic Conditions and Infant Health: A Changing Relationship for Black and White Infants in the United States

**DOI:** 10.1371/journal.pone.0123501

**Published:** 2015-05-14

**Authors:** Chiara Orsini, Mauricio Avendano

**Affiliations:** 1 London School of Economics and Political Science, Cowdray House, Houghton Street, London, WC2A 2AE, United Kingdom; 2 Harvard School of Public Health, Department of Social and Behavioural Sciences, 677 Huntington Avenue, Boston, Massachusetts 02115, United States of America; School of Population Health, The University of Queensland, AUSTRALIA

## Abstract

We study whether the relationship between the state unemployment rate at the time of conception and infant health, infant mortality and maternal characteristics in the United States has changed over the years 1980-2004. We use microdata on births and deaths for years 1980-2004 and find that the relationship between the state unemployment rate at the time of conception and infant mortality and birthweight changes over time and is stronger for blacks than whites. For years 1980-1989 increases in the state unemployment rate are associated with a decline in infant mortality among blacks, an effect driven by mortality from gestational development and birth weight, and complications of placenta while in utero. In contrast, state economic conditions are unrelated to black infant mortality in years 1990-2004 and white infant mortality in any period, although effects vary by cause of death. We explore potential mechanisms for our findings and, including mothers younger than 18 in the analysis, uncover evidence of age-related maternal selection in response to the business cycle. In particular, in years 1980-1989 an increase in the unemployment rate at the time of conception is associated with fewer babies born to young mothers. The magnitude and direction of the relationship between business cycles and infant mortality differs by race and period. Age-related selection into motherhood in response to the business cycle is a possible explanation for this changing relationship.

## Introduction

The sharp downturn in economic activity in many countries in recent years has spurred renewed interest in the relationship between business cycles and health. This relates to a broader literature examining the relationship between business cycles and both economic and non-economic outcomes, with recent research focusing on the relatively unexplored question of whether this relationship changes over time. An intriguing aspect of this literature is that it draws different conclusions depending on the outcomes considered. For example, [1] find that the relationship between economic cycles and labor market outcomes for different demographic groups in the United States are remarkably stable across three decades of time. Also, [2] find that the relationship between cycles and poverty are fairly stable over time. Differently, [3] find a large effect of the reduction in credit supply following the 2008 financial crisis on employment and the decline in inflation adjusted aggregate wages in small firms, but they notice that the relationship between lending supply and economic activity was not evident in the 1997–2007 period.

In this paper, we contribute to this debate by investigating whether the relationship between the unemployment rate at the time of a baby’s conception and infant health in the United States changed over the years 1980–2004 for whites and blacks. A series of studies find that mortality rates decline when unemployment rates rise [4–9]. In particular, there is a seminal paper [10] that shows that for years 1976–1998 higher unemployment at the time of a baby’s conception is associated with lower incidence of low birthweight, fewer congenital malformations and lower postneonatal mortality, findings due to selection –compositional changes in the pool of mothers having babies during downturns –and changes in mothers’ behaviour [10]. Other recent papers have also examined the relationship between unemployment and newborn health (for instance, See [11] for Spain, and [12] for the United States for the period 1989–1999; [12] also studies prenatal care).

More recently, a series of studies has investigated whether the impact of economic cycles on health outcomes changes over time. For instance, recent research [13] finds that elderly mortality was countercyclical for years 1994–2008, contradicting findings of procyclical mortality originally reported by other authors [4]. Importantly, recent research [14] finds that for years 1976–2009 total mortality for adults has shifted from being procyclical at the beginning of the period to being largely unrelated to macroeconomic conditions at the end of the period.

Our paper contributes to the literature on changes in the relationship between business and health along several dimensions. First, we investigate whether the relationship between the unemployment rate at the time of a baby’s conception and babies’ health at birth and infant mortality, established for the period 1976–1998 [10], has changed over the years 1980–2004 for whites and blacks. The United States had a relatively high infant mortality rate and large race disparities in infant mortality in the period under study. Part of the explanation for these disparities might relate to a differential effect of business cycles on infant mortality between blacks and whites. For instance, see [15] for a review of risk factors associated to the gap in mortality between white and black babies as well as information on infant mortality rates for the United States compared to other industrialized countries

Second, we are the first to investigate the relationship between the unemployment rate at the time of a baby’s conception and infant mortality by cause of death, and to examine how this relationship has changed over time for white and black babies. This analysis uncovers that, even if total white infant mortality is not related to the unemployment rate at the time of conception, mortality from different causes of death is. Furthermore, we show that this relationship can differ for whites and blacks depending on the cause and period considered.

Third, we use microdata of linked individual births and infant deaths from the Center of Disease Control and Prevention (CDC). These datasets link several dimensions of health at birth and demographic characteristics of the mother recorded in the birth certificate, most notably race, to the death certificate of the baby. This approach increases precision in the measurement of infant mortality by race, because race is reported by the mother on the birth certificate, whereas race in the death certificate of a deceased infant is reported by the funeral director based on information provided by an informant or by observation.

As opposed to previous research [10], we include births to mothers younger than 18 to examine selection of mothers by age, a potential important explanation for changes in the relationship between business cycles and infant health. We also examine changes over time in the relationship between business cycles and infant health and prenatal care at birth. We find that our results on health outcomes at birth, maternal selection by education and prenatal care are robust to the inclusion of mothers younger than 18. See S1 File, for estimates on the sample of mothers aged 18 or older.

We present estimates of the relationship between the unemployment rate at the time of conception and infant health outcomes, maternal characteristics and prenatal care for sub-periods 1980–1989 and 1990–2004.

## Data and Descriptive Statistics

We use micro data from Linked Birth and Infant Death files provided by the CDC to construct total and cause-specific infant mortality rates for years 1984–1991 and 1996–2004. These data link information from the birth certificate, such as maternal age, race, and maternal education; maternal health behaviours such as prenatal care; and health at birth such as birthweight to information from the death certificate such as age at death and underlying causes of death for each infant under 1 year of age who dies in the United States. Micro data on infant deaths that have been linked to their birth certificate are in the Numerator File (NF) (every year over 97 percent of births are matched to deaths, see S1 File) and micro data on live births are in the Denominator File (DF). We did not include data for more recent years because CDC does not release state identifiers in public-use micro-data files for years 2005 and beyond. Nevertheless, our data cover a 25-year period and enable us to examine whether the relationship between infant mortality and business cycles is stable over this time period. Race, age of the mother, birthweight and prenatal care are reported in all years for all states. Maternal education is available in all years for all states but California and Texas. To keep the sample of states constant for each outcome, we exclude California and Texas for the analysis of maternal education. In the analysis of Apgar scores we also exclude Texas, Connecticut and California, because these states do not report this outcome for all years. We do not analyze data on smoking and drinking while pregnant as these outcomes were not reported by any state before 1989.

CDC did not produce linked birth and infant deaths datasets for years 1980–1983 and 1992–1995. For these years, we use Vital Statistics Natality Files (VSNF) containing birth certificate information for all live births in the United States and the Multiple Causes of Death (MCOD) files containing a unique record of each death occurring in the United States, and information about the decedent’s age, race, gender, place of residence, and cause of death. For years 1981 and 1982, the micro data from MCOD released to the public are not a reliable source to construct mortality rates by cause of death (See: http://www.nber.org/mortality/1981/docs/mort81.pdf), so we use aggregate data on infant mortality by race and cause of death provided by the CDC in the Compressed Mortality File for these two years.

To assign the yearly state unemployment rate in the mother’s state of residence in the year of conception other authors [10] use the date of the last menstrual period reported in the Natality Files. However, the date of the last menstrual period is not reported in the DF, so we use information on the number of weeks of gestation together with the year and month of birth of the child to calculate backwards the time of conception and to assign the yearly unemployment rate to the time of conception. Finally, to study infant health at birth, prenatal care, and selection into motherhood by maternal characteristics we use the DF for years 1989–1991 and 1995–2004 and use VSNF for years 1992–1994 and years 1980–1988, because DF for years 1983–1988 does not have information on the month of birth of the child, which we use to calculate the time of conception. We summarize in S1 File the dataset used in the analysis.

Table 1 reports the 7 most common causes of death and their relative ranking for years 1990 and 2000. Although the ranking differs slightly, we focus on 7 causes of death consistently ranked among the 10 leading causes of infant death for years 1980–2004 in the United States for black and white babies.

**Table 1:** Main Causes of Neonatal and Postneonatal Mortality 1990 and 2000.

Causes of Death	ICD-10 Code (1)	ICD-9 Code (2)	Comparability Ratio (3)	Rank 1990 (4)	Rank 2000 (5)
Congenital malformations	Q00-Q99	740–759	0.9064	1	1
Disorders related to short gestation and low birth weight	P07	765	1.1060	3	2
Sudden infant death syndrome	R95	798.0	1.0362	2	3
Newborn affected by maternal complications of pregnancy	P01	761	1.0295	5	4
Newborn affected by complications of placenta, cord and membranes	P02	762	1.0470	6	5
Respiratory distress of new born	P22	769	1.0257	4	6
Accidents (unintentional injuries)	V01-X59	E800-E869,E880-E929	1.0246	7	7

Source: Anderson et al., 2001 and CDC “Report on infant mortality for the United States for 1990”. Columns 4 and 5 report the ranking of the selected causes of death for years 1990 and 2000.

It is important to highlight that during 1980–2004 two different systems to code causes of death are in place: until 1999 the CDC uses the 9^th^ version of the International Classification of Disease (ICD-9) and for later years the 10^th^ version is used (ICD-10). To achieve comparability between the two classifications, the CDC estimates a comparability ratio calculated by dividing the number of deaths classified in the ICD-10 revision by the number of deaths classified in the ICD-9 revision. The comparability ratios represent the level of correspondence between the ICD-9 and ICD-10 codes for a given condition and a ratio of 1 indicates full comparability between the two codes for a given condition [16]. We report comparability ratios in column 4 of Table 1, which highlights that comparability codes for all seven conditions are close to 1.

We examine infant mortality and health outcomes separately for blacks and whites. Race and Hispanic ethnicity are separately recorded in the birth registries and death certificates. We classify hispanic whites in the white category and hispanic blacks in the black category. S1 File reports summary statistics for blacks and whites for years 1980–2004 and for years 1980–1989 and 1990–2004. On average, there are 3,582,531 births per year from 1980 to 2004. S1 File highlights that for whites and blacks measures of health and prenatal care behaviours improved over time. It also shows that mothers over time tend to be older and of higher socioeconomic status.

## Empirical Strategy

We link data on infant outcomes, maternal characteristics and prenatal care to yearly state unemployment rate data from the Bureau of Labor Statistics and model the relationship between state unemployment rate in the year of conception (the year before death for mortality outcomes) and the outcomes of interest as follows [4, 5, 10]:

$$y_{lt}=U_{st}b+d_{t}+g_{s}+h_{s}\left(g_{s}*t\right)+m\prime X_{st}+v_{lt}$$ (1)

l = s for mortality outcomes, is for health at birth, maternal characteristics and prenatal care.

When studying mortality outcomes we always use a Poisson model so that *y*_*st*_ is the natural logarithm of the infant, neonatal, or postneonatal total or cause-specific mortality rate (per 1000 births) in state of residence s for white and black babies dying in year t. The formula to calculate the outcome is: ln [(number deaths for infants less than one year of age in year t in state s/number of live births in year t in state s)*1000]. Additionally, in Eq 1 *U*_*st*_ is the yearly unemployment rate in maternal state of residence s at time the time of conception; *d*_*t*_ and *g*_*s*_ are year and state fixed effects, respectively; and (*g*_*s*_ * *t*) are state trends. *X*_*st*_ includes the following state level variables, measured at the time of conception: the fraction of the population that is black, the percent of the population covered by health insurance, the percent of the population in poverty. Following [4, 10], for mortality outcomes we weight Eq 1 using the number of births in each state, year and race.

Also, in Eq 1 some outcomes are estimated at the individual birth level. Specifically, in these cases *y*_*ist*_ represents: a dummy equal to 1 if infant i in state s at time t is born with low birthweight (i.e., born with a weight less than 2500 grams); a dummy equal to 1 if infant i in state s at time t is born with very low birthweight (i.e., born with a weight less than 1500 grams); different dummies to identify the different age categories for the mother of child i in state s at time t; different dummies to identify different education categories the mother of child i in state s at time t belongs to; different prenatal care variables for prenatal care of child i in state s at time t. When studying these outcomes we estimate Eq 1 with a linear model. Standard errors are always clustered at the state level.

As previous authors [14], we face the issue of selecting the length, starting and ending dates of sub-periods of analysis. In the main analysis we distinguish periods 1980–1989 and 1990–2004, because both periods contain at least one national recession, allowing us to compare the relationship between state unemployment rates and infant health across a deep contraction (in the early 1980) and two less severe contractions (in the early 1990s and the early 2000s). The starting year of the first period, 1980, and the starting year of the second period, 1990, are the same years used in other work [2] in selecting two of the starting dates of the sub-periods for their study on changes of the cyclicality of poverty (See S1 File for NBER dates of contraction of the national economy), and we follow previous research here [2]. We estimate the following model:

$$y_{lt}=U_{st}b_{8089}D_{8089}+U_{st}b_{9004}D_{9004}+d_{1t}+g_{1s}+h_{1s}(g_{1s}*t)+m_{1}^{\prime }X_{st}+v_{lt}$$ (2)

l = s for mortality outcomes, is for outcomes measuring health at birth, maternal characteristics and prenatal care

Where *D*_8089_ is a dummy equal to 1 for years 1980–1989 and 0 otherwise, and *D*_9004_ is a dummy equal to 1 for years 1990–2004 and 0 otherwise. We test whether *b*_8089_ is equal to *b*_9004_.

We present results from estimating Eq 1 in later sections. Additionally, in the S1 File we present results from estimates of Eq 1 when fixing the time window and changing the starting year of such window. For instance, fixing the time window to ten years we presents graphs plotting estimates of *b* * 100 from Eq 1 for years 1980–1989 and then for years 1981–1990 until years 1995–2004. We also provide graphs of estimates of *b* * 100 from a more basic version of Eq 1 where we do not control for the fraction of the population that is black, the percent of the population covered by health insurance, the percent of the population in poverty and where we estimate a linear model also for mortality outcomes. In this case, we start our analysis with year 1980 and first provide estimates of *b* * 100 from the basic specification of Eq 1 for years 1980–1985, a period that already contains a deep recession and a recovery, and subsequently add one year at a time.

We next modify Eqs 1 and 2 and estimate the following equations for years 1980–2004:

$$y_{lt}=U_{st}b_{w}+U_{st}b_{b}+d_{tw}+d_{tb}+g_{sw}+g_{sb}+h_{sw}\left(g_{sw}*t\right)+h_{sb}\left(g_{sb}*t\right)+m_{b}^{\prime }X_{st}*B+m_{w}^{\prime }X_{st}*W+v_{2lt}$$ (3)

l = s for mortality outcomes, is for health at birth, maternal characteristics and prenatal care.

$$y_{lt}=U_{st}b_{8089w}D_{8089w}+U_{st}b_{9004w}D_{9004w}+U_{st}b_{8089b}D_{8089b}+U_{st}b_{9004b}D_{9004b}+d_{2tw}+d_{2tw}g_{2sw}+d_{2tb}+d_{2tb}g_{2sb}+h_{2sw}\left(g_{2sw}*t\right)+h_{2sb}\left(g_{2sb}*t\right)+m_{2b}^{\prime }X_{st}*B+m_{2w}^{\prime }X_{st}*W+v_{3lt}$$ (4)

l = s for mortality outcomes, is for health at birth, maternal characteristics and prenatal care w and b refer to whites and blacks, respectively. In Eq 3 we test whether we can reject that *b*_*w*_ is equal to *b*_*b*_ for years 1980–2004 and in Eq 4 we test whether we can reject the hypothesis that *b*_8089*w*_ is equal to *b*_8089*b*_ and the hypothesis that *b*_9004*w*_ is equal to *b*_9004*b*_.

## Results

### Infant, Neonatal and Postneonatal Mortality

Depending on the period, our results indicate that an increase in the state unemployment rate is associated with a decline in infant mortality among black babies, while no such relationship is observed for white babies. For the entire period 1980–2004, the point estimates of *b* * 100 in Eq 1 for infant and neonatal mortality (Table 2, rows 1–4) suggest that a higher unemployment rate the year before death is significantly associated with a decline in infant and neonatal mortality for black babies.

**Table 2:** Infant Mortality and Causes of Death.

Outcomes	1980–1989(1)	1990–2004 (2)	1980–2004 (3)	P-Value of F-Test of equality of *b*_8089_ and *b*_9004_ (4)
**1)** Infant Mortality (Whites)	-0.6384% (0.4345) [0.0790]	-0.4549% (0.5231) [0.7597]	-0.5948% (0.4278) [0.1189]	0.6774
**2)** Infant Mortality (Blacks)	-1.8701%*** (0.6398) [0.0790]	-0.6960% (0.9523) [0.7597]	-1.6336%** (0.6518) [0.1189]	0.1862
**3)** Neonatal Mortality (Whites)	-0.5326% (0.4578) [0.0096]	-0.0236% (0.5710) [0.2854]	-0.4148% (0.4706) [0.0225]	0.3082
**4)** Neonatal Mortality (Blacks)	-2.2169%*** (0.7205) [0.0096]	-1.2545% (1.3066) [0.2854]	-2.0262%** (0.8129) [0.0225]	0.2945
**5)** Postneonatal Mortality (Whites)	-0.8365% (0.8246) [0.6558]	-1.4138%* (0.8272) [0.3490]	-0.9684% (0.7481) [0.8150]	0.4357
**6)** Postneonatal Mortality (Blacks)	-1.3718% (0.9573) [0.6558]	-0.4640% (1.0977) [0.3490]	-1.2017% (0.8261) [0.8150]	0.5190
**7)** Mortality for Congenital Malformations, Deformations, and Chromosomal Abnormalities (Whites)	1.9435%*** (0.3931) [0.0256]	0.4410% (0.4447) [0.8749]	1.5707%*** (0.3553) [0.0518]	0.0086
**8)** Mortality for Congenital Malformations, Deformations, and Chromosomal Abnormalities (Blacks)	0.1563% (0.948) [0.0256]	0.2618% (0.97158) [0.8749]	0.1783% (0.85385) [0.0518]	0.9199
**9)** Mortality For Disorders Related to Short Gestation and Low Birth Weight (Whites)	-1.9247%** (0.9671) [0.3545]	-2.4097%** (1.00679) [0.0373]	-2.0716%*** (0.7531) [0.9318]	0.7119
**10)** Mortality For Disorders Related to Short Gestation and Low Birth Weight (Blacks)	-3.2686%** (1.3916) [0.3545]	1.6712% (1.7425) [0.0373]	-1.9445% (1.3726) [0.9318]	0.0144
**11)** Mortality for Sudden Infant Death (Whites)	-2.3105%** (0.91931) [0.9028]	-1.1231% (1.19091) [0.0963]	-1.9936%** (0.95905) [0.5621]	0.2217
**12)** Mortality for Sudden Infant Death (Blacks)	-2.0460% (1.80027) [0.9028]	3.9711% (2.99703) [0.0963]	-0.7830% (1.82471) [0.5621]	0.0209
**13)** Mortality Due to Complications of Placenta, Cord and Membranes, (Whites)	1.1517% (2.12349) [0.0629]	-1.1377% (2.14956) [0.3937]	0.4602% (1.69115) [0.1533]	0.3524
**14)** Mortality Due to Complications of Placenta, Cord and Membranes, (Blacks)	-5.3386%** (2.21142) [0.0629]	1.4424% (2.91957) [0.3937]	-3.5131%* (2.04469) [0.1533]	0.3004

Column 3 reports point estimates of *b* in Eq 1 and its standard errors (in parenthesis) both multiplied per 100.

***, **, and * mean statistical significance at the 1, 5 and 10 percent level, respectively.

In column 1 and 2 are point estimates of *b*_8089_ * 100 and *b*_9004_ * 100 in Eq 2 and their standard errors (in parenthesis). % represents the percent change in the outcome for a one percentage point increase in the state unemployment rate at the time of conception. In square brackets are the P-values of the test of equality of *b*_*w*_ and *b*_*b*_ in Eq 3 (column 3) and P-values of the test of equality of *b*_8089*w*_ and *b*_8089*b*_, and equality between *b*_9004*w*_ and *b*_9004*b*_ in Eq 4 (columns 1 and 2, respectively). Standard errors are clustered at the state level

For whites, higher unemployment is associated with a decline in postneonatal mortality only for years 1990–2004 (Table 2, row 5). P-value of the F-test of the equality of *b*_*w*_ and *b*_*b*_ in Eq 3 in square brackets (Table 2, rows 1–4) indicate that we can reject that the relationship between the unemployment rate the year before death is the same for white and black babies for years 1980–1989 for infant and neonatal mortality.

The relationship between the state unemployment rate and infant and neonatal mortality of black babies weakened between the two periods. In fact, columns 2 and 3 of Table 2 show that there is a stronger relationship between the state unemployment rate and black infant and neonatal mortality in years 1980–1989 compared to years 1990–2004, as *b*_8089_ * 100 is precisely estimated and *b*_9004_ * 100 is not. These results are not driven by sample size, because the second period has more data points than the first period. However, P-values of the F-tests of equality between *b*_8089_ and *b*_9004_ (Table 2, column 4 rows 2 and 4) indicate that we cannot reject the equality between *b*_8089_ and *b*_9004_.

### Mortality from Specific Causes of Death

The heterogeneous analysis of infant mortality by specific causes of death for whites reveals that, despite the lack of a statistically significant relationship between state unemployment rate in the year before death and total white infant mortality, the unemployment rate is associated with mortality from some causes of death. For example, an increase in the state unemployment rate in the year before death is significantly associated with an increase in white mortality for congenital malformations, deformations, and chromosomal abnormalities in years 1980–2004, an effect driven by sub-period 1980–1989. Increased unemployment is also associated with a decline in mortality from disorders related to short gestation and low birthweight in both sub-periods and mortality from sudden infant death in years 1980–1989 (Table 2, rows 7, 9 and 11). For blacks, an increase in the state unemployment rate in the year before death is significantly associated with a decline in infant mortality for disorders related to short gestation and low birthweight, and a decline in mortality due to complications of placenta, cord, and membranes in years 1980–1989 (Table 2, rows 10 and 14). Table 2 reveals that we can reject that the relationship between the state unemployment rate and mortality is the same for whites and blacks during 1980–1989 for congenital malformations, deformations, and chromosomal abnormalities, as well as for mortality from complications of placenta, cord and membranes (Table 2, rows 7). Also, p-values of the F-test of equality between *b*_8089*w*_ and *b*_8089*b*_ in Eq 4, and between *b*_9004*w*_ and *b*_9004*b*_, indicate that we can reject that the relationship between the state unemployment rate and mortality for disorders related to short gestation and low birthweight is the same for blacks and whites in 1990–2004 (Table 2, rows 9–10). Analysis of mortality from other causes of death reveals that only for whites in 1990–2004 an increase in the state unemployment rate is associated with mortality from maternal complications of pregnancy (See S1 File). There is no clear relationship between business cycles and mortality from other causes of death (See S1 File).

### Heath Outcomes at Birth

We study the relationship between the unemployment rate at the time of conception and the fraction of newborns with low birthweight (weight less than 2500 grams) and the fraction of newborns with very low birthweight (weight less than 1500 grams). Birthweight is considered an indicator of infant health, and it is a strong predictor of infant mortality. In addition, disorders related to short gestation and low birthweight were the leading cause of infant death among black infants in the period under study, and the second leading cause of death among white infants. Moreover, children with low birthweight have, on average, worse outcomes in terms of adult health, educational attainment and earnings in adulthood [17, 18], making low birthweight an indicator of negative socio-economic trajectories later in life.

We also carry out analyses on the Apgar scores, a test performed at one and five minutes after birth to evaluate the health of the newborn. We examine the relationship between the state unemployment rate and the fraction of babies with 5 minutes Apgar score lower than 5, a cut-off used in earlier studies [10] that signals poor health outcomes at birth (Table 3, rows 5 and 6).

**Table 3:** Health at Birth.

Outcomes	1980–1989 (1)	1990–2004 (2)	1980–2004 (3)	P-Value of F-Test of equality of *b*_8089_ and *b*_9004_ (4)
**1)** Weight less than 2500 grams (Whites)	-0.02365 (0.01701) -0.42859% [0.0000]	-0.0053 (0.01513) -0.08333% [0.9706]	-0.01775 (0.01126) -0.29339% [0.0010]	0.4547
**2)** Weight less than 1500 grams (Whites)	-0.00447 (0.0072) -0.51379% [0.0001]	0.00515 (0.00469) 0.47247% [0.7039]	-0.00137 (0.0046) -0.13700% [0.0054]	0.2979
**3)** Weight less than 2500 grams (Blacks)	-0.1570*** (0.0437) -1.25099% [0.0000]	-0.0040 (0.0527) -0.03030% [0.9706]	-0.1159*** (0.0333) -0.89498% [0.0010]	0.0209
**4)** Weight less than 1500 grams (Blacks)	-0.0528** (0.0203) -2.0969% [0.0001]	0.0127 (0.0267) 0.4191% [0.7039]	-0.0352** (0.0141) -1.2394% [0.0054]	0.0720
**5)** Apgar score < = 5 (Whites)	0.0046 (0.0066) [0.9751]	0.0032 (0.0062) [0.2564]	0.0041 (0.0048) [0.7287]	0.8742
**6)** Apgar score < = 5 (Blacks)	-0.0008 (0.0223) [0.9751]	0.0192 (0.0179) [0.2564]	0.0049 (0.0154) [0.7287]	0.5009

Column 3 reports point estimates of *b* in Eq 1 and its standard errors (in parenthesis) both multiplied per 100.

***, **, and * mean statistical significance at the 1, 5 and 10 percent level, respectively.

In column 1 and 2 are point estimates of *b*_8089_ * 100 and *b*_9004_ * 100 in Eq 2 and their standard errors (in parenthesis). % represents the percent change in the outcome for a one percentage point increase in the state unemployment rate at the time of conception. In square brackets are the P-values of the test of equality of *b*_*w*_ and *b*_*b*_ in Eq 3 (column 3) and P-values of the test of equality of *b*_8089*w*_ and *b*_8089*b*_, and equality between *b*_9004*w*_ and *b*_9004*b*_ in Eq 4 (columns 1 and 2, respectively). Standard errors are clustered at the state level

Because Apgar scores were not available for Texas, Connecticut and California for all years, we exclude these states from this analysis. This means that our results only apply to the sample of states other than Texas, California and Connecticut.

For white babies, Table 3 (rows 1 and 2) highlights that there is no significant association between the state unemployment rate and the fraction of babies weighting less than 2500 grams and weighting less than1500 grams independently of the period considered.

For black babies, estimates of *b*_8089_ * 100 (Table 3, rows 3 and 4) suggest that there is a significant association between the unemployment rate at conception and the fraction of babies born of low and very low birthweight in the period 1980–1989, while the estimate of *b*_9004_ * 100 shows that there is no such association from 1990 to 2004. The p-value of the F-test of equality of coefficients in Table 3 (column 4, row 3) indicate that we can reject that *b*_8089_ is equal to *b*_9004_ for both birthweight outcomes.

These results point towards differences in the strength of the association between the state unemployment rate at the time of a baby’s conception and birthweight across different periods and for different sub-samples. Results also show that the strength of the relationship between the state unemployment rate at the time of a baby’s conception and the fraction of babies born of low and very low birthweight is different between white and black babies for years 1980–1989 (Table 3, P-values in square brackets).

In summary, the sign of our results when we consider the period 1980–1989 for blacks is in line with the sign of the results for years 1976–1998 of [10] on the sample of mothers aged 18 or more. However, for blacks we do not find a statistically significant association between the state unemployment rate at the time of conception and birthweight outcomes for the sub-period 1990 to 2004. For white babies, our finding that an increase in the unemployment rate at the time of conception is unrelated to birthweight outcomes is in line with findings by [10] for years 1976–1998.

## Mechanisms

### Maternal age

The medical literature suggests a U-shaped relationship between maternal age and infant mortality and the likelihood for a baby to be born with low birthweight [19–23]. Evidence from the United States [20] and Sweden [23] suggests that maternal ages below 18 and above 35 increase the likelihood of adverse birth outcomes. In this analysis, we focus on the following outcomes: the fraction of mothers aged 18 or less, the fraction of mothers aged between 18 and 24, the fraction of mothers aged 25 or less, the fraction of mothers between 25 and 35, and the fraction of mother older than 35. Table 4 (rows 1–5) shows that estimates of *b*_9004_ * 100 from Eq 2 are statistically significant for whites when the outcomes are the fraction of mothers aged between 18 and 24 and the fraction of mothers older than 35 years of age.

**Table 4:** Characteristics of Mothers.

Outcomes	1980–1989 (1)	1990–2004 (2)	1980–2004 (3)	P-Value of F-Test of equality of *b*_8089_ and *b*_9004_ (4)
**1)** Mother younger than 18 (Whites)	-0.03463* (0.01755) -0.92101% [0.9290]	0.01197 (0.0298) 0.34297% [0.1866]	-0.0196 (0.0122) -0.54596% [0.4278]	0.2350
**2)** Mother between 18 and 24, (Whites)	0.0167 (.0259) 0.04488% [0.8771]	0.0739* (.0415) 0.25200% [0.2951]	0.0351 (.0265) 0.10890% [0.5431]	0.1764
**3)** Mother younger than 25 (Whites)	-0.2674*** (0.0922) -0.65260% [0.9058]	0.0550 (0.1745) 0.16760% [0.2474]	-0.1637** (0.0819) 0.45710% [0.3886]	0.1064
**4)** Mother between 25 and 35, (Whites)	0.2596*** (0.0883) 0.47710% [0.9449]	0.0103 (0.156) 0.01980% [0.4623]	0.1794** (0.0763) 0.3399% [0.5911]	0.1623
**5)** Mother older than 35 (Whites)	0.0078 (0.0178) 0.16919% [0.7302]	-.06538* (0.0388) -0.4256% [0.0105]	-0.0156 (0.0213) -0.1368% [0.2090]	0.0561
**6)** Mother younger than 18 (Blacks)	-0.0407 (0.0383) -0.3772% [0.9290]	-0.0697 (0.0477) -0.08065% [0.1866]	-0.0485 (0.0316) -0.5143% [0.4278]	0.6190
**7)** Mother between 18 and 24, (Blacks)	0.0291 (0.0386) 0.0618% [0.8771]	-0.0279 (0.0608) -0.0679% [0.2951]	0.0137 (0.0377) 0.03166% [0.5431]	0.3464
**8)** Mother younger than 25 (Blacks)	-0.2487** (0.1121) -0.4296% [0.9058]	-0.2460 (0.2095) -0.4948% [0.2474]	-0.2480*** (0.0840) -0.4705% [0.3886]	0.9920
**9)** Mother between 25 and 35, (Blacks)	0.2447** (0.1153) 0.6425% [0.9449]	0.1815 (0.1947) 0.4786% [0.4623]	0.2277*** (0.0819) 0.5968% [0.5911]	0.8059
**10)** Mother older than 35 (Blacks)	0.00401 (0.0133) 0.1129% [0.7302]	0.0645 (0.0403) 0.5218% [0.0105]	0.0202 (0.0174) 0.2142% [0.2090]	0.1079
**11)** Mother has less than high school (Whites)	-0.1479* (0.0857) -0.8113% [0.1618]	0.2566 (0.1492) 1.34768% [0.9723]	-0.0154 (0.0855) -0.08209% [0.1648]	0.0096
**12)** Mother has high school (Whites)	-0.0027 (0.1128) -0.0063% [0.0715]	0.5511*** (0.2020) 1.6920% [0.1876]	0.1785** (0.085) 0.49418% [0.9491]	0.0366
**13)** Mother with more than high school (Whites)	0.1507 (0.1787) 0.3860% [0.8832]	-0.8077** (0.3237) -1.6694% [0.5061]	-0.1631 (0.1523) 0.3615% [0.3557]	0.0165
**14)** Mother with less than high school (Blacks)	-0.2915*** (0.1010) 0.8876% [0.1618]	0.2483 (0.3331) 0.9042% [0.9723]	-0.1415 (0.1383) 0.4823% [0.1648]	0.0925
**15)** Mother with high school (Blacks)	0.1160 (0.1348) 0.2706% [0.0715]	0.3091 (0.2945) 0.7813% [0.1876]	0.1697 (0.1329) 0.4168% [0.9491]	0.5393
**16)** Mothers with more than high school (Blacks)	0.1754 (0.1908) 0.7221% [0.8832]	-0.5574 (0.5635) -1.6901% [0.5061]	-0.0281 (0.2441) 0.0938% [0.3557]	0.1720

Column 3 reports point estimates of *b* in Eq 1 and its standard errors (in parenthesis) both multiplied per 100.

***, **, and * mean statistical significance at the 1, 5 and 10 percent level, respectively.

In column 1 and 2 are point estimates of *b*_8089_ * 100 and *b*_9004_ * 100 in Eq 2 and their standard errors (in parenthesis). % represents the percent change in the outcome for a one percentage point increase in the state unemployment rate at the time of conception. In square brackets are the P-values of the test of equality of *b*_*w*_ and *b*_*b*_ in Eq 3 (column 3) and P-values of the test of equality of *b*_8089*w*_ and *b*_8089*b*_, and equality between *b*_9004*w*_ and *b*_9004*b*_ in Eq 4 (columns 1 and 2, respectively). Standard errors are clustered at the state level

In contrast, estimates of *b*_8089_ * 100 (Table 4, rows 1–5) are precisely estimated when the outcomes are the fraction of mothers younger than 18, the fraction of mothers younger than 25, and the fraction of mothers between 25 and 35. Specifically, white mothers are less likely to be less than 18 and less than 25 and are more likely to be between 25 and 35 years of age when unemployment rates rise in the period 1980–1989. P-values of the F-Test of equality of coefficients *b*_8089_ and *b*_9004_ (Table 4, column 4, row 5) show that we can reject that *b*_8089_ is equal to *b*_9004_ when the outcome is the fraction of white mothers older than 35. Also, we cannot reject that the relationship between the unemployment rate and maternal age is different for periods 1980–1989 and 1990–2004 when the outcome is the fraction of mothers younger than 25 (P-value is 0.1064).

The increase in the fraction of mothers who are prime-aged (namely between 25 and 35 years of age) when unemployment rates rise for years 1980–1989 is a possible explanation for the decline in white infant mortality for disorders related to short gestation and low birth weight and the decline in infant mortality for sudden infant death [24]. By contrast, the increase in maternal age could be responsible for the increase in white infant mortality for congenital malformations, deformations and chromosomal abnormalities which increases when unemployment rates rise in years 1980–1989. Also, in years 1990–2004 the decline in the fraction of mothers older than 35 and the increase in the fraction of mothers between 18 and 24 in periods of relatively high unemployment are possible explanations for the decline in mortality from disorders related to short gestation and low birth weight in 1990–2004 for whites.

Similarly, black mothers tend to be older when unemployment rates rise (Table 4, rows 6–10), but again this relationship differs by period. In fact, *b* * 100 from Eq 1 and *b*_8089_ * 100 from Eq 2 are precisely estimated when the outcomes are the fraction of mothers less than 25 years of age and the fraction of mothers aged between 25 and 35, but *b*_9004_ * 100 is not precisely estimated, results pointing towards a stronger relationship for years 1980–1989 compared to years 1990–2004. Selection into motherhood by age is different for blacks and whites for years 1980–2004 when the outcome is the fraction of mothers older than 35 (See P-values in square brackets of F-Test of equality of coefficients *b*_*w*_ and *b*_*b*_ in Table 4, rows 1–10, column 3).

The increase in the fraction of black mothers who are prime-aged (25–35) in periods of relatively high unemployment in years 1980–1989 may explain the decline in mortality from disorders related to short gestation and low birthweight and mortality for complications of placenta, cord, and membranes.

Our results are partially different from results by [10] for years 1976–1998 for their sample of mothers aged 18 or older. In fact, in their specification with state trends, [10] find that when unemployment rates rise white mothers are less likely to be younger than 25 an older than 35, and they are instead more likely to be between 25 and 35. They also find that black mothers are less likely to be younger than 25, more likely to be between 25 and 35, and older than 35 when unemployment rises. Our results show that maternal selection by age during economic cycles depends on the period under study and the sample considered.

### Maternal Education

For whites, we reject that the relationship between the state unemployment rates at the time of conception and mother’s education is the same for years 1980–1989 and years 1990–2004 (see P-values of the F-Tests of equality of *b*_8089_ and *b*_9004_ of Eq 3 in Table 4, column 4, rows 11–13). For years 1990–2004 only, an increase in the state unemployment rate at the time of conception is associated with an increase in the fraction of mothers who have high school and a decline in the fraction of white mothers who have more than high school. The finding that in years 1990–2004 white mothers are negatively selected when unemployment rates rise is in line with results reported by [10] for years 1978–1998. However, our results suggest that the choice of the period matters, as we find no evidence of negative selection by maternal education for years 1980–1989.

For blacks, we find that for years 1980–1989 an increase in the state unemployment rate at the time of conception is associated with a decline in the fraction of mothers with less than high school, but there is no evidence of positive selection by education in periods of relatively high employment for years 1990–2004 (Table 4, rows 13–16). For blacks we reject that the relationship between the state unemployment rate at the time of conception and the fraction of mothers with education lower than high school is the same in years 1980–1989 and years 1990–2004 (See P-values of the F-Tests of equality of *b*_8089_ and *b*_9004_ in Table 4, column 4, row 14). Our results differ from those by [10] because, although we find that blacks are positively selected in terms of educational outcomes in periods of relatively high unemployment, this result only holds for years 1980–1989 and not for years 1990–2004.

Analysis on maternal education excluded data from California and Texas, as data on education was not available for all years in these states. This means that our analysis on maternal selection by education only applies to the sample of states other than California and Texas. In summary, this section has the aim to show that, when keeping the sample of states constant over time, maternal selection by education changes over time.

### Prenatal Care

We consider the following outcomes: the average number of prenatal care visits, the fraction of babies with less than 5 prenatal care visits, and the fraction of babies who received prenatal care in the first trimester of gestation.

For whites, an increase in the state unemployment rate is associated with a decline in the fraction of babies receiving sub-optimal prenatal care as measured by the fraction of babies with less than 5 visits of prenatal care in years 1990–2004 (Table 5, rows 1–3).

**Table 5:** Prenatal Care.

Outcomes	1980–1989 (1)	1990–2004 (2)	1980–2004 (3)	P-Value of F-Test of equality of *b*_8089_ and *b*_9004_ (4)
**1)** Average number of prenatal care visits (Whites)	0.34915 (1.90745) 0.0316% [0.6228]	2.54742 (1.63374) 0.2191% [0.0093]	1.08262 (1.47754) 0.0947% [0.2562]	0.3441
**2)** Less than 5 prenatal care visits (Whites)	0.09257 (0.11584) 2.3494% [0.0316]	-0.2330*** (0.0835) -4.5956% [0.1173]	-0.01607 (0.0609) 0.3763% [0.0319]	0.0762
**3)** Prenatal care in the first trimester (Whites)	0.15375 (0.15925) 0.1927% [0.5082]	-0.09859 (0.09795) -0.1179% [0.0015]	0.07355 (0.10469) 0.0895% [0.0700]	0.1743
**4)** Average number of prenatal care visits (Blacks)	1.4236 (2.2766) 0.1511% [0.6228]	8.3947*** (2.8754) 0.8018% [0.0093]	3.3225 (2.3619) 0.3292% [0.2562]	0.0068
**5)** Less than 5 prenatal care visits (Blacks)	-0.2004 (0.1464) -1.4294% [0.0316]	-0.4776*** (0.1508) -4.7617% [0.1173]	-0.2759** (0.1369) -2.4075% [0.0319]	0.0537
**6)** Prenatal care in the first trimester (Blacks)	0.2413 (0.1451) 0.3924% [0.5082]	0.4301** (0.1626) 0.6115% [0.0015]	0.2906** (0.1348) 0.4334% [0.0700]	0.2613

Column 3 reports point estimates of *b* in Eq 1 and its standard errors (in parenthesis) both multiplied per 100.

***, **, and * mean statistical significance at the 1, 5 and 10 percent level, respectively.

In column 1 and 2 are point estimates of *b*_8089_ * 100 and *b*_9004_ * 100 in Eq 2 and their standard errors (in parenthesis). % represents the percent change in the outcome for a one percentage point increase in the state unemployment rate at the time of conception. In square brackets are the P-values of the test of equality of *b*_*w*_ and *b*_*b*_ in Eq 3 (column 3) and P-values of the test of equality of *b*_8089*w*_ and *b*_8089*b*_, and equality between *b*_9004*w*_ and *b*_9004*b*_ in Eq 4 (columns 1 and 2, respectively). Standard errors are clustered at the state level

For whites, we can reject that the relationship between the state unemployment rate and the fraction of babies with less than 5 visits of prenatal care is the same for years 1980–1989 and years 1990–2004 (Table 5, row 2, column 4). The decline in the fraction of babies with substandard prenatal care for whites in periods of high unemployment is a possible mechanism behind the decline in infant mortality for disorders related to short gestation and low birthweight in years 1990–2004.

The sign of our estimate of the relationship between the state unemployment rate and the fraction of white babies with less than 5 visits of prenatal care for years 1990–2004 is consistent with the sign found by [10] for years 1976–1998. However- unlike [10]-we find that the state unemployment rate has not significant association with the average number of prenatal care visits for whites.

For blacks, an increase in the state unemployment rate is associated with an increase in the average number of prenatal care visits and with a decline in the fraction of babies with less than 5 visits of prenatal care in years 1990–2004. We also find that when unemployment rates rise black babies are more likely to receive prenatal care in the first trimester in years 1990–2004 (Table 5, row 6).

## Conclusion

We investigate whether the relationship between the unemployment rate at the time of conception and infant mortality, health at birth, maternal characteristics and prenatal care changes during years 1980–2004 for blacks and whites. Our results show that estimates of the relationship between unemployment rate at the time of conception and infant health, maternal characteristics and prenatal care vary depending on the period considered as well as for whites and blacks.

We divide years 1980–2004 into two sub-periods and find that the state unemployment rate is more strongly related to infant health outcomes at birth and maternal characteristics in years 1980–1989 than in years 1990–2004. We also find that outcomes for blacks are more likely to respond to changes in the unemployment rate at the time of conception than outcomes for whites. Prenatal care is more sensitive to changes in the unemployment rate at conception in years 1990–2004 compared to years 1980–1989. For instance, in years 1990–2004, when unemployment rates rise, white and black babies are less likely to have less than 5 visits of prenatal care.

The lack of a relationship between the state unemployment rate at the time of conception and infant mortality for whites for years 1980–1989 and years 1990–2004 masks important heterogeneities. In fact, white infant mortality for disorders related to short gestation and low birthweight and infant mortality for sudden infant death decline when unemployment rates rise, whereas white infant mortality for malformations, deformations and congenital abnormalities increases for years 1980–1989. An increase in the fraction of white mothers who are prime-aged with respect to fertility (namely are between 25 and 35) when unemployment rates rise is a possible reason for the decline in white infant mortality for disorders related to short gestation and low birthweight for years 1980–1989. Similarly, the decline in the fraction of babies with less than 5 visits of prenatal care when unemployment rates rise is a possible mechanism behind the decline in white infant mortality for disorders related to short gestation and low birthweight for years 1990–2004.

For blacks, we find that the decline in infant, neonatal and postneonatal mortality when unemployment rates rise in years 1980–1989 is driven by declines in infant mortality from disorders related to short gestation and low birthwright and declines in infant mortality due to complications of placenta, cord, and membranes. Possible explanations for our findings are the increase in the fraction of black mothers who are of prime-aged with respect to fertility (namely between 25 and 35) in years 1980–1989 when unemployment rates rise.

Our results can be interpreted within the conceptual framework provided by [10] for analysing fertility selection and behavioural change in relation to the business cycle. Within this framework, a decline in women’s wages in periods of downturns (holding other household income constant) generates an income and substitution effect. In general, a decline in women’s wages would lead to a decrease in the demand for children (income effect). On the other hand, according to Becker’s framework [25], children are time intensive so that a decrease in wage income when unemployment rises would lower the relative cost of children, thus increasing the demand for children (substitution effect). The net prediction is ambiguous and depends on the relative magnitudes of the income and substitution effects. Differently, a decline in wages of other household members does not affect the value of time of a woman and therefore has an unambiguous income effect (which implies a decline in the demand for children). However, whether the income effect dominates the substitution effect is ultimately an empirical question and the relative importance of the two effects may differ for different demographic groups and over time. In fact, our results on maternal selection by age over the business cycle show that the relative importance of the income and substitution effects can vary for different groups and over time. For example, the increase in the relative size of the group of mothers between 25 and 35 in periods of relatively high unemployment in 1980–1989 suggests that the relative importance of the substitution effect is larger for this group of mothers compared to the relative importance of the substation effect for mothers of other ages, but this conclusion does not apply to years 1990–2004.

Also, the framework by [10] suggests that a decrease in wages will also have both income and substitution effects on health-related behaviour, including the use of prenatal care services. In fact, the income effect implies lower consumption of all goods, including prenatal care. As an example, consider the case of transportation. If during downturns some mothers cannot afford a car, then it becomes more difficult for them to reach a doctor’s office to get prenatal care and this could imply a decrease in prenatal care in periods of downturns for this group of mothers. On the other hand, health related activities are time intensive and therefore we expect that a decline in wages will lead to substitution into healthier behaviours, due to a reduction in the opportunity costs of time. This, for example, could mean that some mothers, who could not find time to get prenatal care in periods of booms, may be more likely to use prenatal care in periods of downturns. The above examples suggest that the impact of booms and busts on healthy behaviour can be heterogeneous for different groups. In principle, the relative importance of income and substitution effect can also vary over time, making it an empirical question to determine which effect dominates for a given demographic group in a given period of time. Within this framework, our finding that prenatal care increases when unemployment rates rise in years 1990–2004 but not in years 1980–1989 is consistent with an increase in the relative importance of the substitution effect compared to the income effect over time.

In summary, we find that the association between economic conditions and infant mortality changes over time and differs by race. Our findings are in line with recent literature showing that total mortality has shifted from being pro-cyclical in earlier decades to being largely unrelated to macroeconomic conditions in recent years [14]. Whether infant mortality is pro-cyclical, countercyclical or a-cyclical depends on race, cause of death and the period examined.

## Supporting Information

S1 File Supporting information. (PDF)
