# Supplementary material for: Macro-Economic Conditions and Infant Health: A Changing Relationship for Black and White Infants in the United States
Source: PLoS One. 2015 May 14;10(5):e0123501. doi: 10.1371/journal.pone.0123501 (PMC4431876; doi:10.1371/journal.pone.0123501)
Supplement: S1 File — (PDF) [file pone.0123501.s001.pdf]

Supporting Information

Macro-economic Conditions and Infant Health: a Changing Relationship for Black and White Infants in The Unites States

1. Tables

Table A. Datasets Used in the Analysis

	Years					
**Panel A**	**1980**	**1981-1982**	**1983**	**1984-1991**	**1992-1995**	**1996-2004**
**Infant, Neonatal and Postneonatal Mortality and Mortality From Specific Causes of Death**	Vital Statistics Natality Files and Multiple Causes of Death Files	Vital Statistics Natality Files and Compressed Mortality Files	Vital Statistics Natality Files and Multiple Causes of Death Files	Linked Birth and Infant Death Files	Vital Statistics Natality Files and Multiple Causes of Death Files	Linked Birth and Infant Death Files

	Years			
**Panel B**	**1980-1988**	**1989-1991**	**1992-1994**	**1995-2004**
**Health Outcomes at Birth, Selection by Maternal Characteristics and Prenatal Care**	Vital Statistics Natality Files	Denominator File of the Linked Birth and Infant Death Files	Vital Statistics Natality Files	Denominator File of the Linked Birth and Infant Death Files

Table B. Summary Statistics

		Whites			Blacks	
	**(1)** **1980-1989**	**(2)** **1980-2004**	**(3)** **1980-2004**	**(4)** **1980-1989**	**(5)** **1990-2004**	**(6)** **1980-2004**
**1)** Ln(Infant Mortality)	2.2047 (0.1488)	1.8329 (0.1755)	1.9816 (0.246)	2.942 (0.2982)	2.6535 (0.3363)	2.7694 (0.3512)
**2)** Ln(Neonatal Mortality)	1.8441 (0.2908)	1.3935 (0.1795)	1.4995 (0.2253)	2.6129 (0.4041)	2.2343 (0.3353)	2.334 (0.2242)
**3)** Ln (Postneonatal Mortality)	1.3689 (0.5175)	0.7692 (0.3156)	0.8619 (0.2888)	2.1278 (0.6137)	1.6175 (0.4699)	1.6551 (0.2591)
**4)** Ln (Mortality for Congenital Malformations, Deformations, and Chromosomal Abnormalities)	0.6872 (0.2173)	0.3692 (0.2737)	0.4966 (0.2968)	0.8361 (0.5253)	0.5794 (0.4816)	0.6829 (0.5151)
**5)** Ln (Mortality for Disorders Related to Short Gestation and Low Birth Weight)	-0.5733 (0.4618)	-0.4293 (0.4396)	-0.4869 (0.4539)	0.807 (0.5256)	0.9839 (0.5485)	0.9144 (0.5463)
**6)** Ln (Mortality for Sudden Infant Death)	0.1169 (0.4862)	-0.3461 (0.5672)	-0.1597 (0.582)	0.9119 (0.6206)	0.5274 (0.7000)	0.6842 (0.6947)
**7)** Ln (Mortality Due to Complications of Placenta, Cord and Membranes)	-1.5316 (0.4908)	-1.539 (0.5009)	-1.5363 (0.4968)	-0.7234 (0.8175)	-0.6225 (0.7118)	-0.6625 (0.7566)
**8)** Mother younger than 25	0.4097 (0.4918)	0.3281 (0.4695)	0.3581 (0.4794)	0.5789 (0.4937)	0.4971 (0.4999)	0.527 (0.4993)
**9)** Mother between 25 and 35	0.5441 (0.498)	0.5183 (0.4997)	0.5278 (0.4992)	0.3855 (0.4867)	0.3792 (0.4852)	0.3815 (0.4858)
**10)** Mother older than 35	0.0461 (0.2098)	0.1536 (0.3605)	0.1140 (0.3178)	0.0355 (0.1852)	0.1236 (0.3292)	0.0915 (0.2883)
**11)** Mother younger than 18	0.0376 (0.1903)	0.0349 (0.1835)	0.0359 (0.186)	0.1079 (0.3104)	0.08642 (0.281)	0.0943 (0.2922)
**12)** Mother between 18 and 24	0.3721 (0.4833)	0.2932 (0.4552)	0.3222 (0.4673)	0.4709 (0.4991)	0.4107 (0.4919)	0.4327 (0.4954)
**13)** Mother with less than high school, all	0.1823 (0.3861)	0.1904 (0.3927)	0.1876 (0.3904)	0.3284 (0.4696)	0.2746 (0.4463)	0.2934 (0.4553)
**14)** Mother with less than high school, 18 or older	0.1552 (0.3621)	0.1655 (0.3716)	0.1619 (0.3684)	0.2556 (0.4362)	0.2132 (0.4096)	0.2278 (0.4194)
**15)** Mother with high school, all	0.4273 (0.4947)	0.3257 (0.4686)	0.3612 (0.4803)	0.4286 (0.4949)	0.3956 (0.4889)	0.4071 (0.4913)
**16)** Mother with high school, 18 or older	0.4399 (0.4964)	0.3341 (0.4717)	0.371 (0.4831)	0.4715 (0.49919)	0.4265 (0.4946)	0.442 (0.4966)
**17)** Mother with more than high school, all	0.3904 (0.4878)	0.4838 (0.4997)	0.4512 (0.4976)	0.2429 (0.4289)	0.3298 (0.4701)	0.2995 (0.458)

**18)** Mother with more than high school, 18 or older	0.4049 (0.4909)	0.5004 (0.4999)	0.4671 (0.4989)	0.2728 (0.4454)	0.3602 (0.4801)	0.3302 (0.4703)
**19)** Apgar Score less than 5	0.0088 (0.0932)	0.0069 (0.0828)	0.0076 (0.0868)	0.0197 (0.1391)	0.0155 (0.1237)	0.0171 (0.1295)
**20)** Weight less than 2500 grams, all	0.05518 (0.2283)	0.0636 (0.244)	0.0605 (0.2384)	0.1255 (0.3312)	0.1319 (0.3384)	0.1295 (0.3358)
**21**) Weight less than 2500 grams, mother 18 or older	0.0541 (0.2262)	0.0627 (0.2425)	0.0596 (0.2367)	0.1240 (0.3296)	0.1311 (0.3375)	0.128 (0.3347)
**22)** Weight less than 1500 grams, all	0.0087 (0.0927)	0.0109 (0.1037)	0.0100 (0.0998)	0.0252 (0.1567)	0.0303 (0.1714)	0.0284 (0.1662)
**23)**Weight less than 1500 grams, mother 18 or older	0.0084 (0.0913)	0.0106 (0.1027)	0.00983 (0.0987)	0.02494 (0.1559)	0.0303 (0.1714)	0.0284 (0.166)
**24)** Average number of prenatal care visits, all	11.047 (3.8077)	11.626 (3.9251)	11.4234 (3.8942)	9.4176 (4.519)	10.469 (4.619)	10.0926 (6.6111)
**25)** Average number of prenatal care visits, mothers 18 or older	11.117 (3.7724)	11.6732 (3.9044)	11.479 (3.8679)	9.5527 (4.5174)	10.5666 (4.6197)	10.209 (4.6094)
**26)** Less than 5 prenatal care visits, all	0.0394 (0.1945)	0.0507 (0.2194)	0.0427 (0.2021)	0.1402 (0.3472)	0.1003 (0.3004)	0.1146 (0.3186)
**27)** Less than 5 prenatal care visits, mothers 18 or older	0.0477 (0.2128)	0.0365 (0.1876)	0.0404 (0.1969)	0.1337 (0.3403)	0.0970 (0.296)	0.1099 (0.3128)
**28)** Prenatal care in the first trimester, all	0.7976 (0.4018)	0.8359 (0.3703)	0.8216 (0.3828)	0.6152 (0.4865)	0.7034 (0.4567)	0.6705 (0.4700)
**29)** Prenatal care in the first trimester, mothers 18 or older	0.8045 (0.3966)	0.8422 (0.3645)	0.8278 (0.3775)	0.6368 (0.4809)	0.7189 (0.4495)	0.6888 (0.4630)

Means and standard deviations are in parenthesis. Variables in natural logarithm are weighted by the number of births in each state. For variables other than mortality outcomes and the average number of prenatal care visits the Table reports the fraction of observations that belong to the group specified by the dependent variable and the sample (for example, in row 28, column 1, the fraction of mothers who had prenatal care in the first trimester is 0.7976 and such fraction is estimated on the entire sample of mothers).

Table C: Specific Causes of Death, Estimation Results

**Outcomes**	**1980-1989 (1)**	**1990-2004 (2)**	**1980-2004 (3)**	**P-Value of F-Test of equality of b_{8089} and b_{9004} (4)**
**1)** Newborn affected by Maternal Complications of Pregnancy (Whites)	-2.0421% (3.5846) [0.8041]	5.1063%** (2.4744) [0.3873]	0.0775% (3.1704) [0.4697]	0.0680
**2)** Newborn affected by Maternal Complications of Pregnancy (Blacks)	-2.7378% (2.7779) [0.8041]	1.9592% (4.7559) [0.3873]	-1.5556% (2.8454) [0.4697]	0.3004
**3)** Accidents (Whites)	-2.8373% (2.4375) [0.8092]	2.7764% (2.2595) [0.3829]	-1.2761% (1.9192) [0.5634]	0.1321
**4)** Accidents (Blacks)	-3.47825% (2.9442) [0.8092]	-0.00781% (2.3017) [0.3829]	-2.6816% (2.4590) [0.5634]	0.3156
**5)** Bacterial Sepsis of the Newborn (Whites)	0.2530% (0.9979) [0.4592]	-1.0549% (1.8370) [0.7433]	0.0440% (1.0464) [0.4986]	0.3322
**6)** Bacterial Sepsis of the Newborn (Blacks)	0.9644% (1.2542) [0.4592]	-0.3356% (1.8946) [0.7433]	0.7619% (1.2027) [0.4986]	0.4673

Column 3 reports point estimates of b in equation 1 in the main text and its standard errors (in parenthesis) both multiplied per 100. ***, **, and * mean statistical significance at the 1, 5 and 10 percent level, respectively. In column 1 and 2 are point estimates of $b_{8089} * 100$ and $b_{9004} * 100$ in equation 2 in the main text and their standard errors (in parenthesis). The numbers with % represent the percentage change in the outcome when the state unemployment rate at the time of conception (or the year before death for mortality outcomes) increases by one percentage point. In square brackets in column 3 there are the p-values of the test of equality of b_w and b_b in equation 3 in the main text. In square brackets in column 1 and 2 there are the P-values of the test of equality of b_{8089w} and b_{8089b} , and equality between b_{9004w} and b_{9004b} in equation 4 in the main text, respectively. Standard errors are clustered at the state level. Data are weighted by the number of births in each state for each race.

Table D. Health at Birth, Prenatal Care and Mother's characteristics, Excluding mothers younger than 18

**Outcomes**	**1980-1989**<br>**(1)**	**1990-2004**<br>**(2)**	**1980-2004**<br>**(3)**	**P-Value of F-Test of equality of b_{8089} and b_{9004}**<br>**(4)**
**1)** Weight less than 2500 grams (Whites)	-0.0271<br>(0.0166)<br>-0.5009%<br>[0.0000]	0.0014<br>(0.0147)<br>0.0223%<br>[0.7966]	-0.0018<br>(0.0107)<br>-0.0302%<br>[0.0007]	0.2294
**2)** Weight less than 1500 grams (Whites)	-0.0054<br>(0.0073)<br>-0.6428%<br>[0.0001]	0.0064<br>(0.0046)<br>0.6038%<br>[0.6196]	-0.0015<br>(0.0046)<br>-0.1526%<br>[0.0026]	0.1972
**3)** Weight less than 2500 grams (Blacks)	-0.1838***<br>(0.04652)<br>-1.4822%<br>[0.0000]	0.0056<br>(0.05624)<br>0.0427%<br>[0.7966]	-0.1317***<br>(0.0352)<br>-1.0289%<br>[0.0007]	0.0064
**4)** Weight less than 1500 Grams (Blacks)	-0.0618***<br>(0.0220)<br>-2.4778%<br>[0.0001]	0.0202<br>(0.0280)<br>0.6666%<br>[0.6196]	-0.0392**<br>(0.0151)<br>-1.3803%<br>[0.0026]	0.0312
**5)** Mother with less than high school (Whites)	-0.1262<br>(0.0823)<br>-0.8131%<br>[0.1176]	0.2711*<br>(0.1386)<br>-0.7625%<br>[0.9856]	0.0045<br>(0.0798)<br>0.0278%<br>[0.1044]	0.0083
**6)** Mother with high school (Whites)	-0.0187<br>(0.1159)<br>-0.0425%<br>[0.0554]	0.5519**<br>(0.2051)<br>1.6519%<br>[0.2232]	0.1690*<br>(0.0872)<br>0.4555%<br>[0.9305]	0.0346
**7)** Mother with more than high school (Whites)	0.1450<br>(0.1797)<br>0.3581%<br>[0.7803]	-0.8230**<br>(0.3201)<br>-1.6447%<br>[0.4985]	-0.1735<br>(0.1493)<br>-0.3714%<br>[0.3269]	0.0161
**8)** Mother with less than high school (Blacks)	-0.3144**<br>(0.1283)<br>-1.2301%<br>[0.1176]	0.2952<br>(0.275)<br>1.3846%<br>[0.9856]	-0.1405<br>(0.1349)<br>-0.6167%<br>[0.1044]	0.0282
**9)** Mother with high school (Blacks)	0.1326<br>(0.1533)<br>0.2812%<br>[0.0554]	0.2898<br>(0.3253)<br>0.6795%<br>[0.2232]	0.1774<br>(0.1562)<br>0.4013%<br>[0.9305]	0.6391
**10)** Mothers with more than high school (Blacks)	0.1817<br>(0.2050)<br>0.6661%<br>[0.7803]	-0.5851<br>(0.5443)<br>-1.6244%<br>[0.4985]	-0.0369<br>(0.2405)<br>-0.1117%<br>[0.3269]	0.1508
**11)** Average number of prenatal care visits (Whites)	0.4513<br>(1.8490)<br>0.0406%<br>[0.5789]	2.4203<br>(1.6148)<br>0.2073%<br>[0.0094]	1.1109<br>(1.4378)<br>0.0968%<br>[0.2329]	0.3835
**12)** Less than 5 prenatal care visits (Whites)	0.0904<br>(0.1112)<br>1.8952%	-0.2193***<br>(0.0799)<br>-6.0082%	-0.0133<br>(0.0581)<br>0.3292%	0.0788

	[0.0213]	[0.0912]	[0.0208]	
**13)** Prenatal care in the first trimester (Whites)	0.1602<br>(0.1536)<br>0.1991%<br>[0.4425]	-0.1056<br>(0.0984)<br>-0.1254%<br>[0.0014]	0.0753<br>(0.0997)<br>0.0909%<br>[0.0539]	0.1487
**14)** Average number of prenatal care visits (Blacks)	1.69886<br>(2.31549)<br>[0.5789]<br>0.1778%	8.22597***<br>(2.87992)<br>[0.0094]<br>0.7785%	3.52277<br>(2.39209)<br>[0.2329]<br>0.3451%	0.0101
**15)** Less than 5 prenatal care visits (Blacks)	-0.22828<br>(0.14684)<br>-1.7074%<br>[0.0213]	-0.47417***<br>(0.15092)<br>-4.8883%<br>[0.0912]	-0.29699**<br>(0.13674)<br>2.7024%<br>[0.0208]	0.0865
**16)** Prenatal care in the first trimester (Blacks)	0.27277*<br>(0.14419)<br>0.4283%<br>[0.4425]	0.4322***<br>(0.15896)<br>0.6012%<br>[0.0014]	0.31544**<br>(0.13227)<br>0.4579%<br>[0.0539]	0.3463

Column 3 reports point estimates of b in equation 1 in the main text and its standard errors (in parenthesis) both multiplied per 100. ***, **, and * mean statistical significance at the 1, 5 and 10 percent level, respectively. In column 1 and 2 are point estimates of $b_{8089} * 100$ and $b_{9004} * 100$ in equation 2 in the main text and their standard errors (in parenthesis). The numbers with % represent the percentage change in the outcome when the state unemployment rate at the time of conception (or the year before death for mortality outcomes) increases by one percentage point. In square brackets in column 3 there are the p-values of the test of equality of b_w and b_b in equation 3 in the main text. In square brackets in column 1 and 2 there are the P-values of the test of equality of b_{8089w} and b_{8089b} , and equality between b_{9004w} and b_{9004b} in equation 4 in the main text, respectively. Standard errors are clustered at the state level. Data are weighted by the number of births in each state for each race.

2.NBER periods of national contractions, years 1980-2004

1/1980-7/1980

7/1981-11/1982

7/1990-3/1991

3/2001-11/2001

3. Fraction of birth certificates linked to the death certificates by year

All deaths were linked to their birth certificate for years 1984-1986. 97.8 percent of deaths were linked in 1987, 97.2 percent in 1988, 97.4 percent in 1989, 97.5 percent in 1990, 97.7 in 1991, 97.8 in 1996, 97.9 in 1997, 98.3 in 1998, 98.1 in 1999, 98.7 in 2000, 99 in 2001, 2002 and in 2003, 98.9 in 2004, 98.7 in 2005, 98.7 in 2006, 98.4 in 2007, 98.7 in 2008, 98.6 for 2009

4. Estimates of a linear model with different time windows

Figures A-F report point estimates of $b * 100$ from the following Equation:

$$y_{lt} = U_{st}b + d_t + g_s + h_s(g_s * t) + v_{lt} \qquad \text{(A)}$$

l=s for mortality outcomes, is for health at birth, maternal characteristics and prenatal care.

Where y_{st} represents the natural logarithm of the infant, neonatal, or postneonatal total or cause-specific mortality rate (per 1000 births)[1] in state of residence s for white and black babies dying in year t; U_{st} is the yearly unemployment rate in maternal state of residence s at time the time of conception t; d_t and g_s are year and state fixed effects, respectively; and $(g_s * t)$ are state trends. Following Rhum (2000; 2013) and Dehejia and Lleras-Muney (2004), for mortality outcomes we weight equation 1 using the number of births in each state, year and race. y_{it} represents health outcomes at birth, mother's characteristics and prenatal care behaviour. We estimate equation A using a linear model and cluster the standard errors at the state level. We start our analysis with year 1980 and first estimate equation A on years 1980-1985, a period that already contains a deep recession and a recovery, and subsequently add one year at a time and provide all estimates of equation A in graphs in Figures A-F below.

[1] The formula to calculate the outcome is: ln [(number deaths for infants less than one year of age in year t in state s/number of births in year t in state s)*1000] for the natural logarithm of the mortality rates.

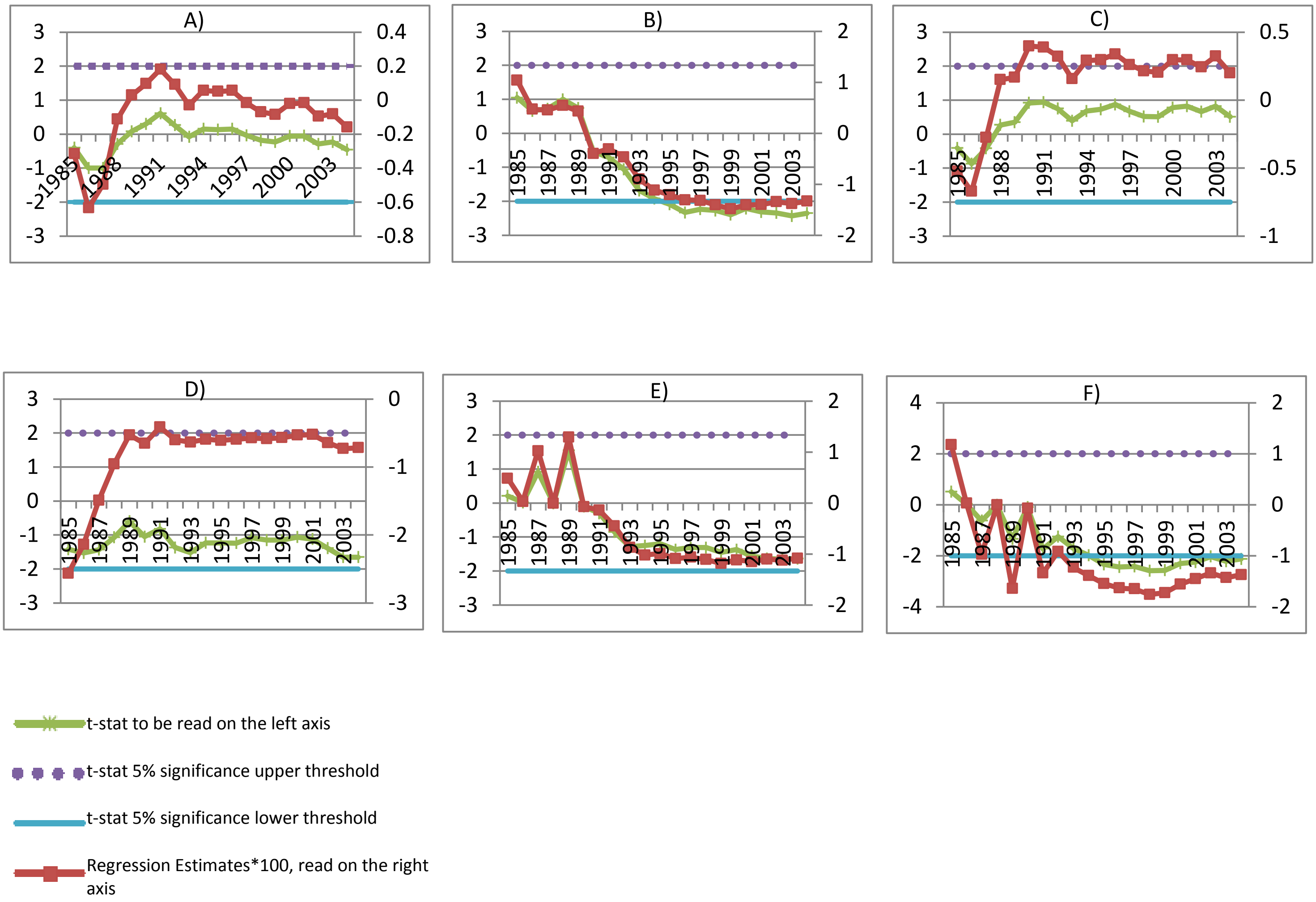

Figure A. A) White Infant mortality, Different Periods,1980-2004; B) Black Infant Mortality, Different Periods, 1980-2004; C) White Neonatal Mortality, Different Periods, 1980-2004; D) White Postneonatal Mortality, Different Periods, 1980-2004; E) Black Neonatal Mortality, Different Periods, 1980-2004; F) Black Postneonatal Mortality, Different Periods, 1980-2004. Figures reports point estimates of $b * 100$ in Equation A and its associated t-statistics for different periods for several infant mortality outcomes. The first data point is for years 1980-1985, the second is for years 1980-1986, and so on until 1980-2004.

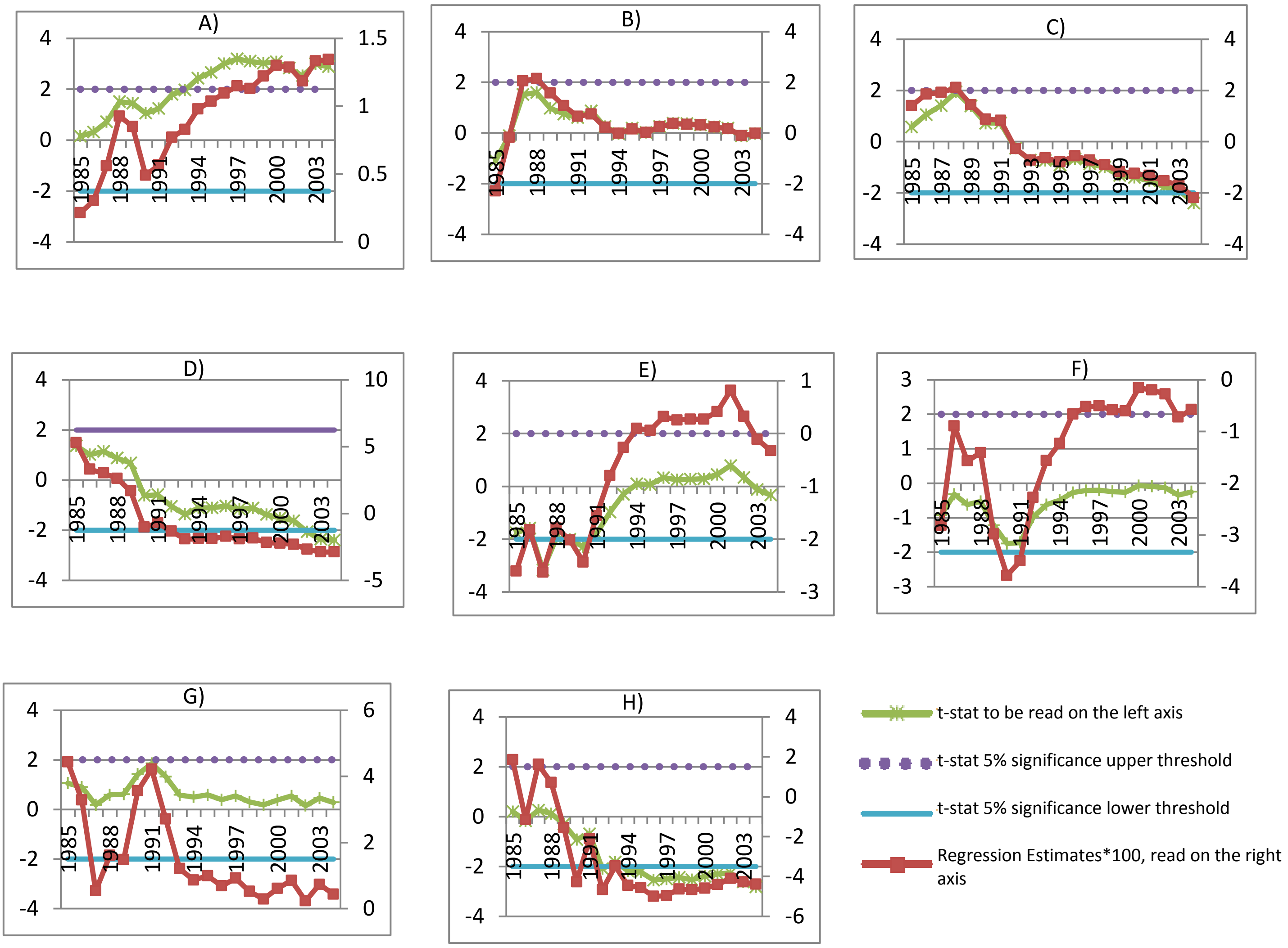

Figure B. A)White Infant Mortality For Congenital Malformations, Deformations and Chromosomal Abnormalities, Different Periods, 1980-2004; B) Black Infant Mortality For Congenital Malformations, Deformations and Chromosomal Abnormalities, Different Periods, 1980-2004; C) White Infant Mortality For Disorders Related to Short Gestation and Low Birthweight, Different Periods, 1980-2004; D) Black Infant Mortality For Disorders Related to Short Gestation and Low Birthweight, Different Periods, 1980-2004; E) White Infant Mortality For Sudden Infant Death, Different Periods, 1980-2004; F) Black Infant Mortality For Sudden Infant Death, Different Periods, 1980-2004; G)White Infant Mortality due to Complications of Placenta, Cord, and Membranes; H) Black Infant Mortality due to Complications of Placenta, Cord, and Membranes, Different Periods 1980-2004. Figures reports point estimates of $b * 100$ in Equation A) and its associated t-statistics for different periods for several infant mortality outcomes. The first data point is for years 1980-1985, the second is for years 1980-1986, and so on until 1980-2004.

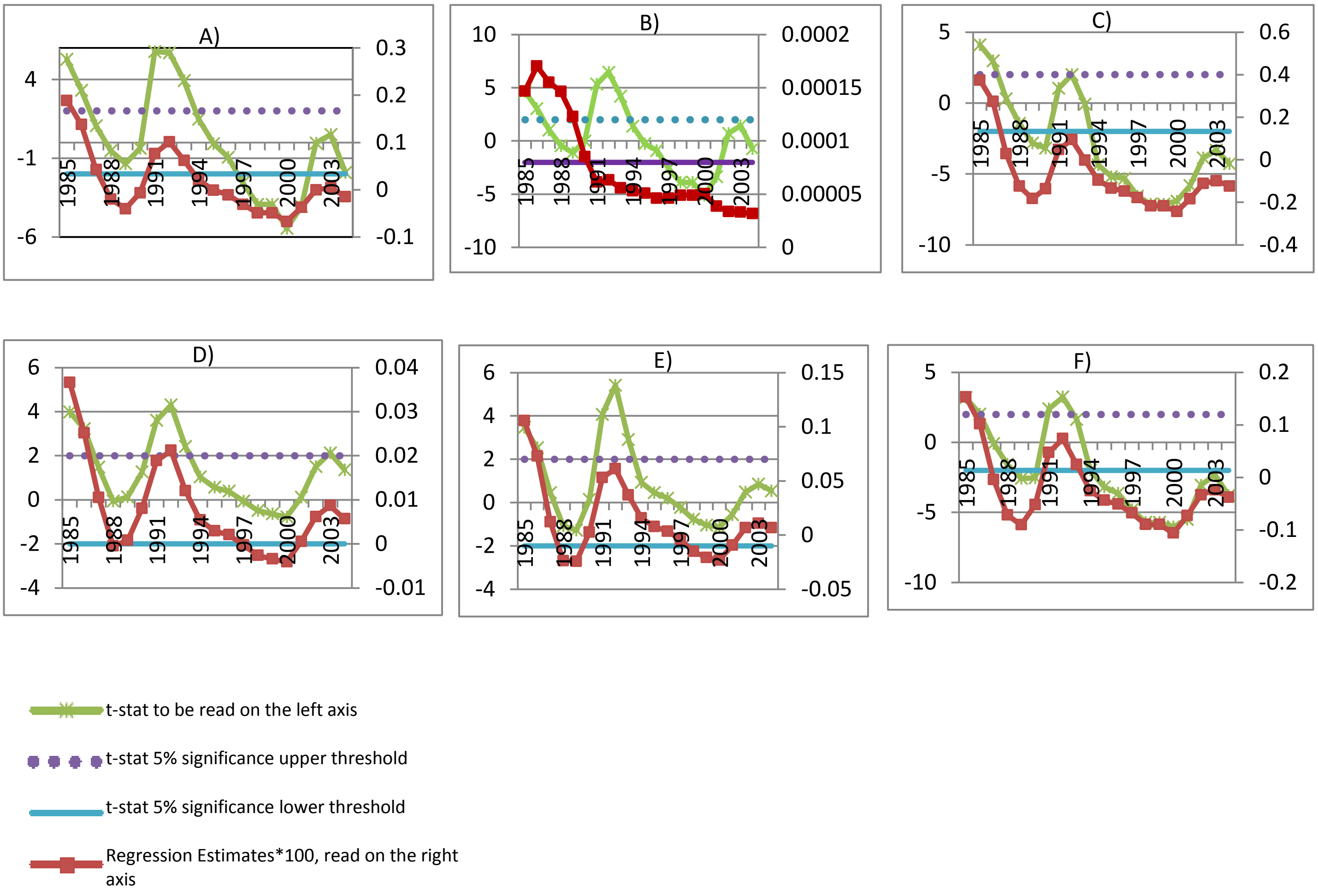

Figure C. A) Fraction of White Babies who weight less than 2500 Grams, Different Periods,1980-2004; B) Fraction of White Babies who weight less than 2500 grams, Different Periods, 1980-2004; C) Fraction of Black Babies who weight less than 2500 grams, Different Periods, 1980-2004; D) Fraction of Black Babies who weight less than 1500 Grams, Different Periods, 1980-2004; E) Fraction of White Babies with 5-min Apgar Score less than or Equal to 5, Different Periods, 1980-2004; F) Fraction of Black Babies with 5-min Apgar Score Less than or equal to 5, Different Periods, 1980-2004. Figures report point estimates of $b * 100$ in Equation A and its associated t-statistics for different periods for health outcomes at birth. The first data point is for years 1980-1985, the second is for years 1980-1986, and so on until 1980-2004.

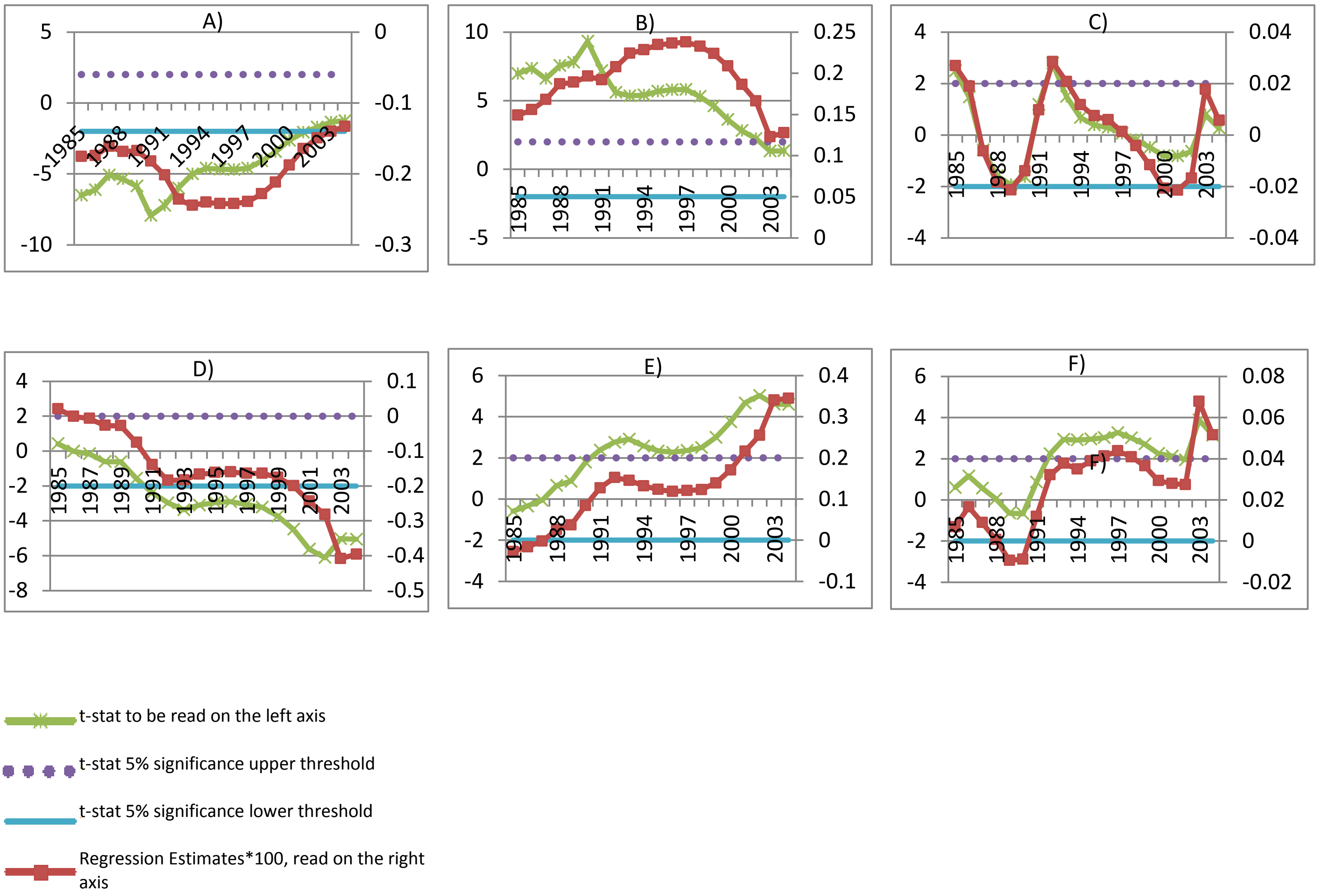

Figure D. A) Fraction of White Mothers Aged Less than 25, Different Periods, 1980-2004; B) Fraction of White Mothers Aged Between 25 and 35, Different Periods, 1980-2004; C) Fraction of White Mothers Aged more than 35, Different Periods, 1980-2004; D) Fraction of Black Mothers Aged less than 25, Different Periods, 1980-2004; E) Fraction of Black Mothers Aged Between 25 and 35, Different Periods, 1980-2004; F) Fraction of Black Mothers Older than 35, Different Periods, 1980-2004. Figures report point estimates of $b * 100$ in Equation A and its associated t statistics for different periods when the outcomes are selected maternal characteristics. The first data point is for years 1980-1985, the second is for years 1980-1986, and so on until 1980-2004.

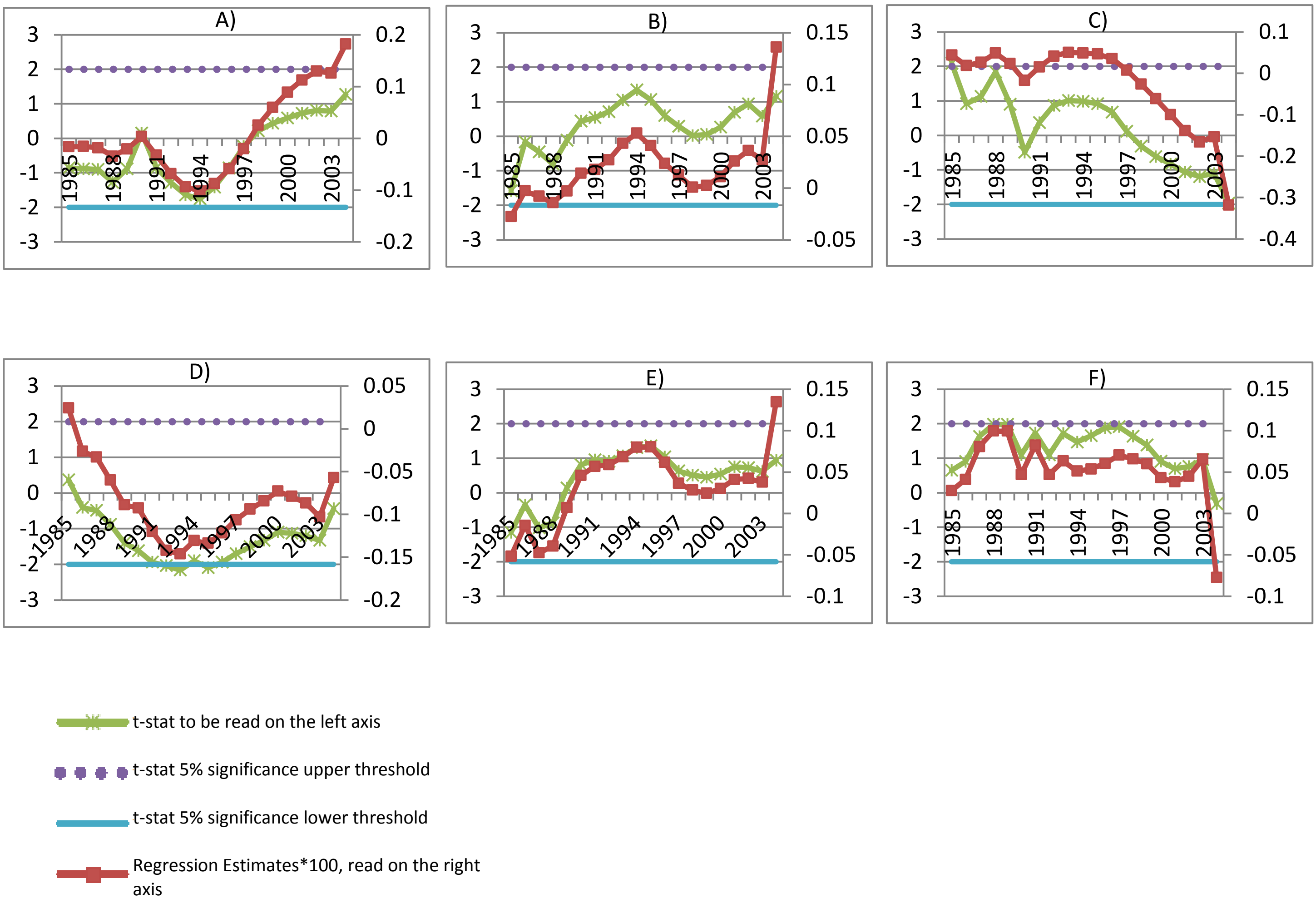

Figure E.A) Fraction of White Mothers with level of education lower than high school, Different Periods, 1980-2004; B) Fraction of White Mothers with level of education equal to high school, Different Periods, 1980-2004; C) Fraction of White Mothers with level of education higher than high school, Different Periods, 1980-2004; D) Fraction of Black Mothers with level of education lower than high school, Different Periods, 1980-2004; E) Fraction of Black Mothers with level of education higher than high school, Different Periods, 1980-2004; F) Fraction of Black Mothers with level of education higher than high school, Different Periods, 1980-2004. Figures report point estimates of $b * 100$ in Equation A and its associated t statistics for different periods when the outcomes are selected maternal characteristics. The first data point is for years 1980-1985, the second is for years 1980-1986, and so on until 1980-2004.

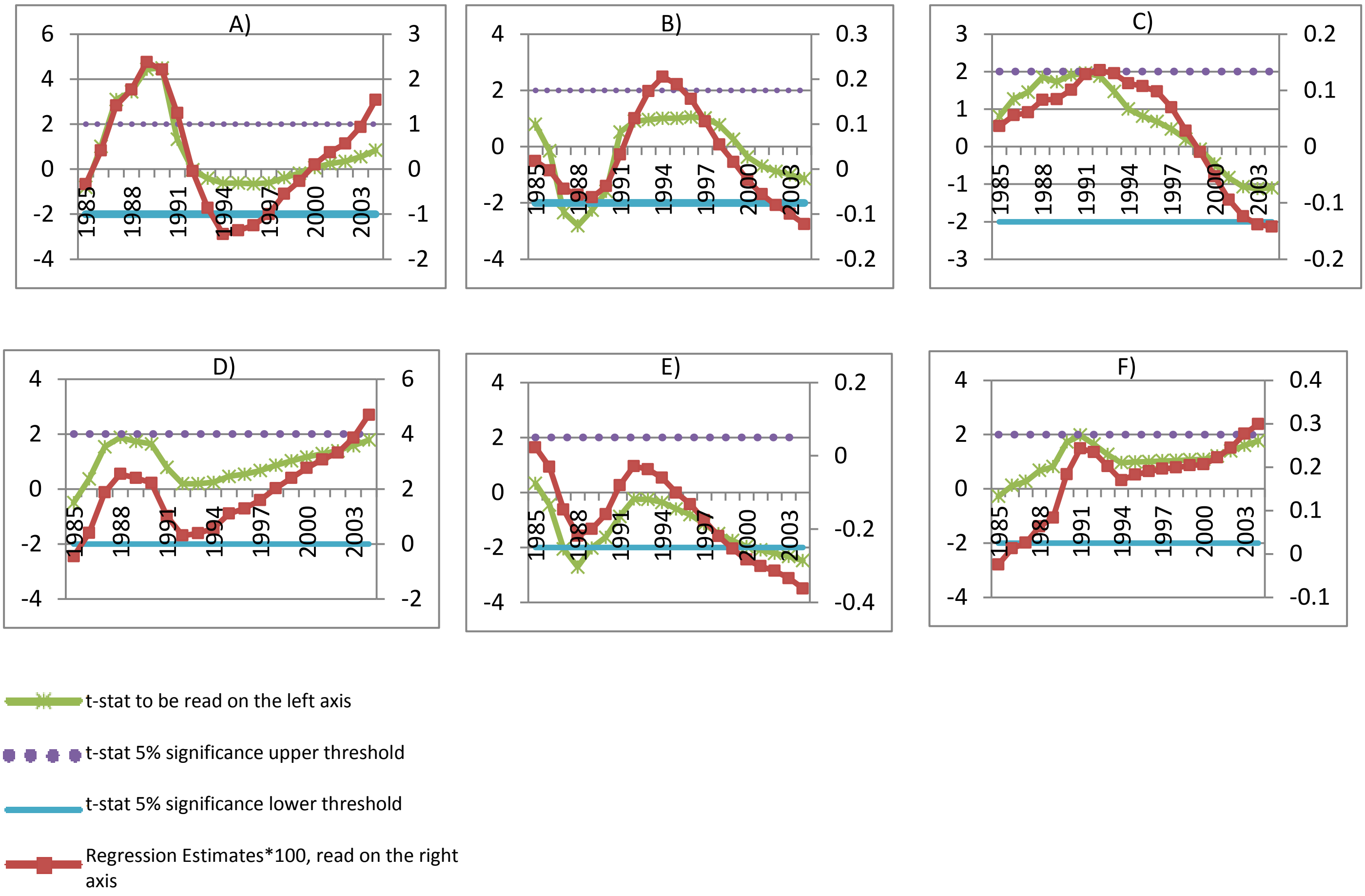

Figure F. A) Average Number of Prenatal Care visits, White Mothers, Different Periods, 1980-2004; B) Fraction of White Mothers With Less than 5 Prenatal Care Visits, Different Periods, 1980-2004; C) Fraction of White Mothers With Prenatal Care in the First Trimester, Different Periods, 1980-2004; D) Average Number of Prenatal Care visits, Black Mothers, Different Periods, 1980-2004; E) Fraction of Black Mothers With less than 5 Prenatal Care Visits, Different Periods, 1980-2004; F) Fraction of Black Mothers With Prenatal Care in the First Trimester, Different Periods, 1980-2004. Figures report point estimates of $b * 100$ in Equation A and its associated t statistics for different periods when the outcomes are selected prenatal care outcomes. The first data point is for years 1980-1985, the second is for years 1980-1986, and so on until 1980-2004.

5. Estimates with fixed time windows

Figures G-M report point estimates of $b * 100$ from equation 1 in the main text fixing the time window for each estimate to 10 year intervals. So, for instance, the first point estimates is on data for the 10 years between 1980 and 1989, the second point estimate is for years 1981-1990 and so on until the last point estimate that is for years 1995-2004. In Figure M the time window is 8 years.

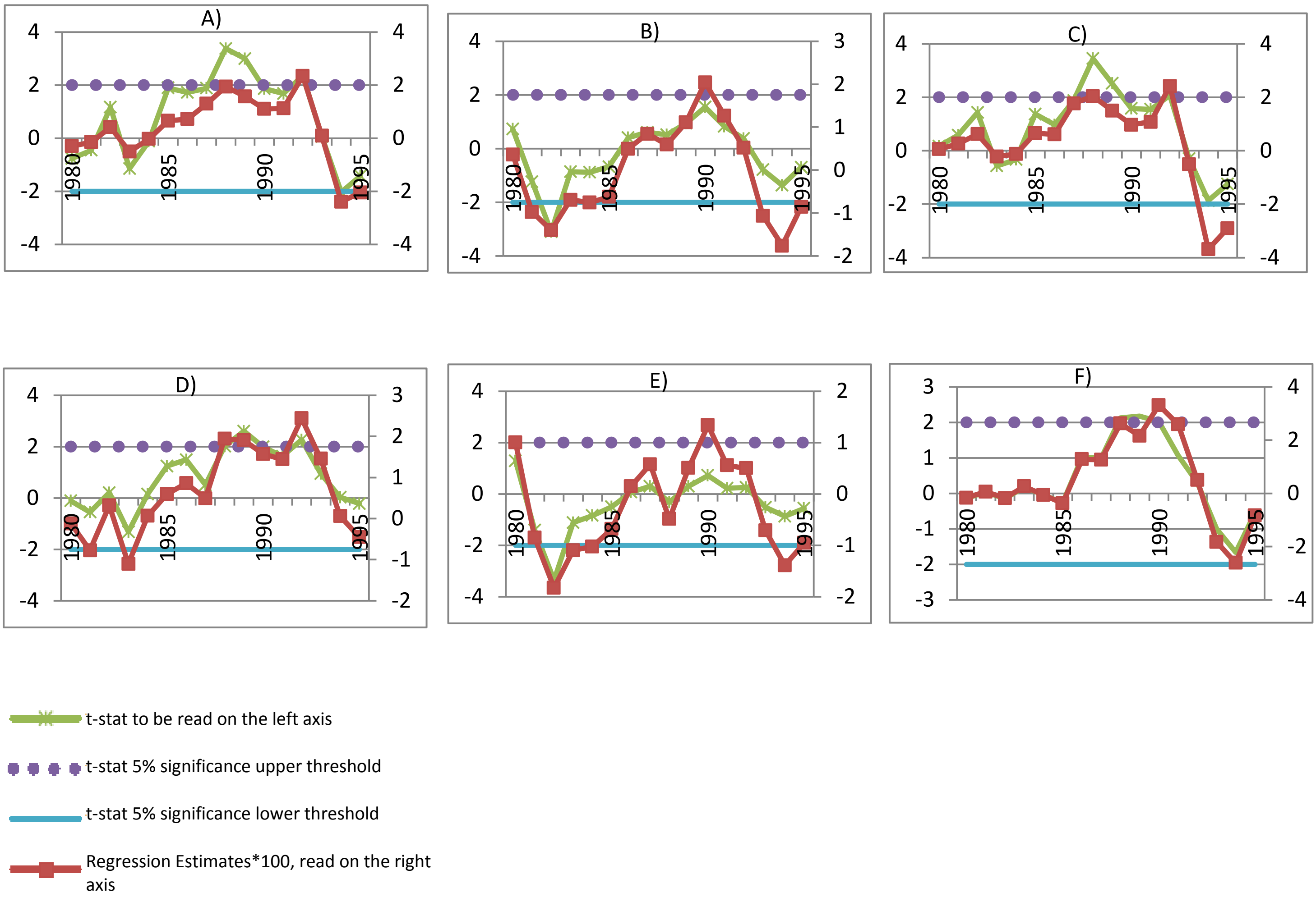

Figure G. A) White Infant Mortality,Different Periods, 1980-2004; B) Black Infant Mortality, Different Periods, 1980-2004; C) White Infant Mortality, Different Periods, 1980-2004; D) White Postnenonatal Mortality, Different Motality, Different Periods, 1980-2004; E) Black Neonatal Motality, Different Periods, 1980-2004; F) Black Postneonatal Mortality, Different Periods, 1980-2004. Figures report point estimates of $b * 100$ in equation 1 in the main text and its associated t-statistics for different periods for several infant mortality outcomes. The first data point is for years 1980-1989, the second is for years 1981-1990, and so on until 1995-2004.

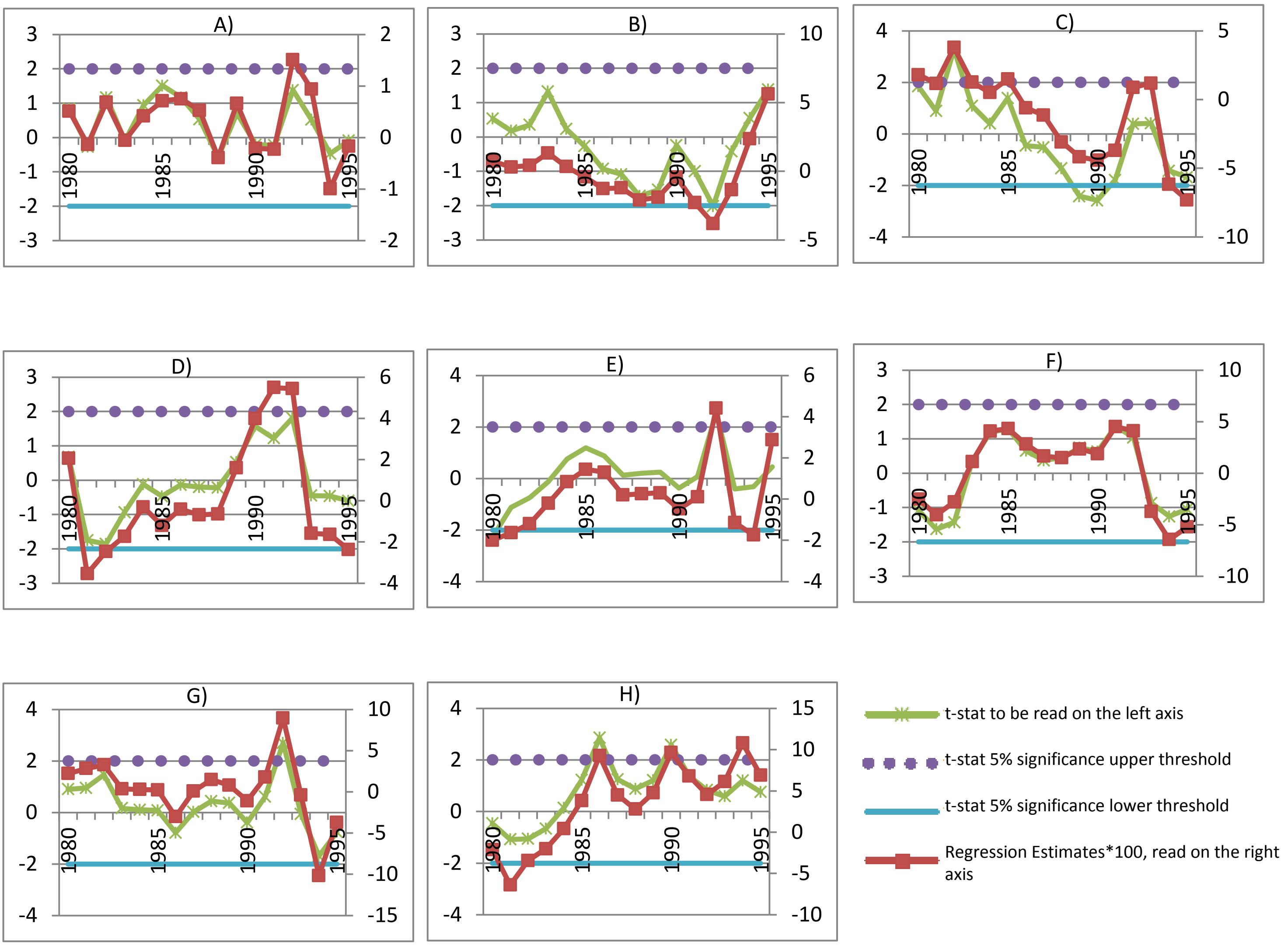

Figure H. A) White Infant Mortality For Congenital Malformations, Deformations and Chromosomal Abnormalities, Different Periods, 1980-2004; B) Black Infant Mortality For Congenital Malformations, Deformations and Chromosomal Abnormalities, Different Periods, 1980-2004; C) White Infant Mortality For Disorders Related to Short Gestation and Low Birthweight, Different Periods, 1980-2004; D) Black Infant Mortality For Disorders Related to Short Gestation and Low Birthweight, Different Periods, 1980-2004; E) White Infant Mortality For Sudden Infant Death, Different Periods, 1980-2004; F) Black Infant Mortality For Sudden Infant Death, Different Periods, 1980-2004; G) White Infant Mortality due to Complications of Placenta, Cord, and Membranes; H) Black Infant Mortality due to Complications of Placenta, Cord, and Membranes, Different Periods 1980-2004. Figures report point estimates of $b * 100$ in equation 1 in the main text and its associated t-statistics for different periods for several infant mortality outcomes. The first data point is for years 1980-1989, the second is for years 1981-1990, and so on until 1995-2004.

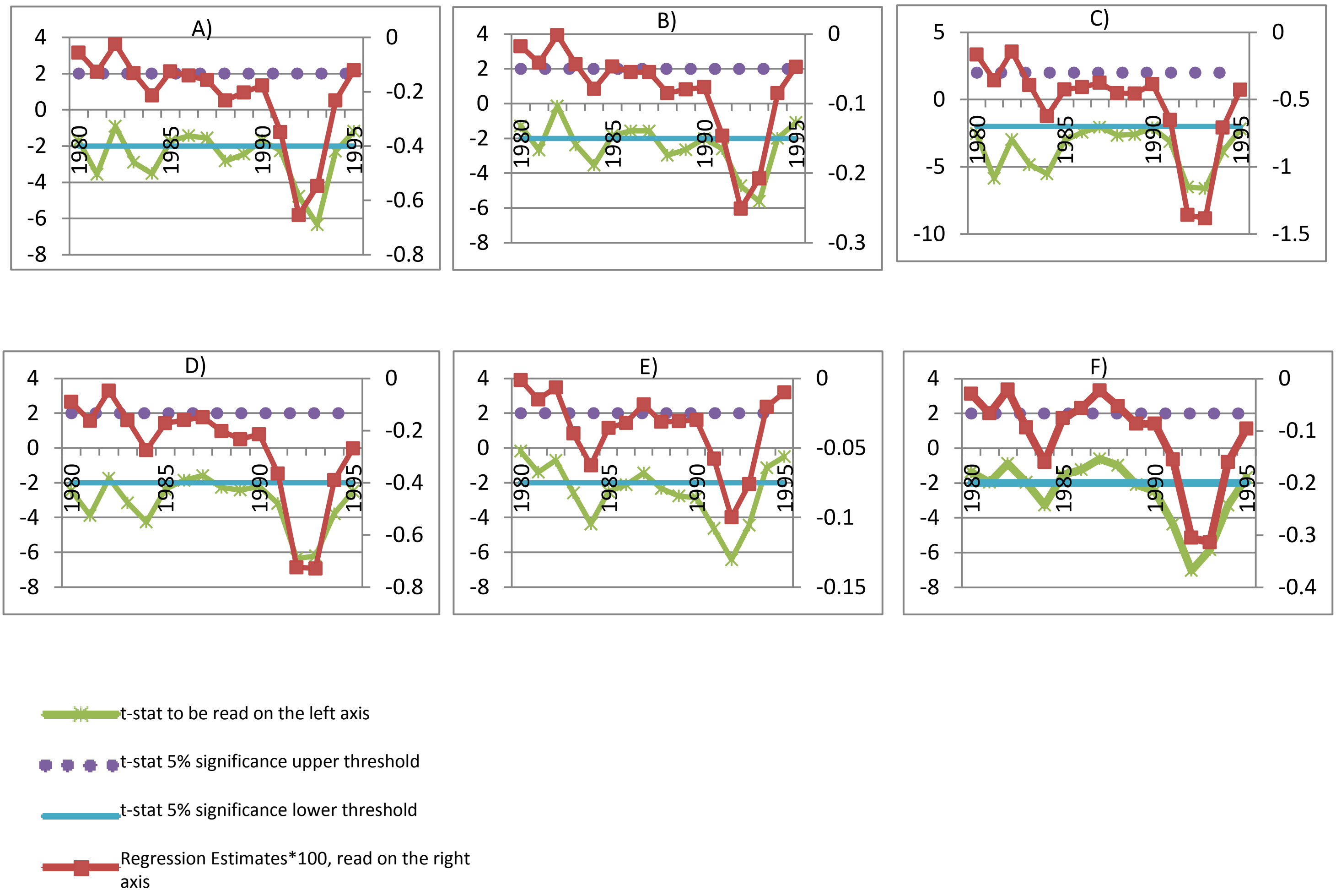

Figure I. A) Fraction of White Babies who weight less than 2500 Grams, Different Periods,1980-2004; B) Fraction of White Babies who weight less than 2500 grams, Different Periods, 1980-2004; C) Fraction of Black Babies who weight less than 2500 grams, Different Periods, 1980-2004; D) Fraction of Black Babies who weight less than 1500 Grams, Different Periods, 1980-2004; E) Fraction of White Babies with 5-min Apgar Score less than or Equal to 5, Different Periods, 1980-2004; F) Fraction of Black Babies with 5-min Apgar Score Less than or equal to 5, Different Periods, 1980-2004. Figures report point estimates of $b * 100$ in equation 1 in the main text and its associated t-statistics for different periods for several infant mortality outcomes. The first data point is for years 1980-1989, the second is for years 1981-1990, and so on until 1995-2004.

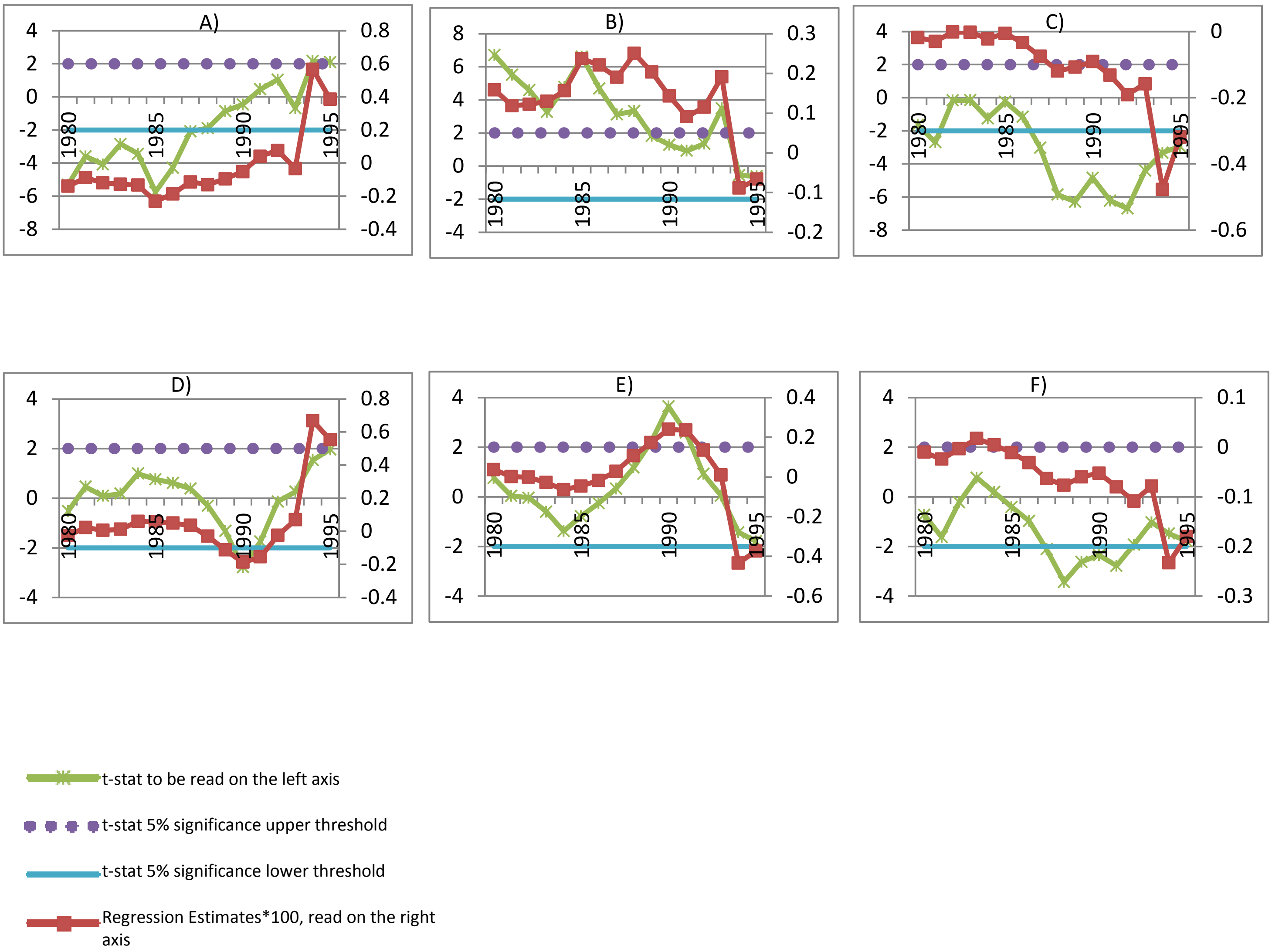

Figure J. A) Fraction of White Mothers Aged Less than 25, Different Periods, 1980-2004; B) Fraction of White Mothers Aged Between 25 and 35, Different Periods, 1980-2004; C) Fraction of White Mothers Aged more than 35, Different Periods, 1980-2004; D) Fraction of Black Mothers Aged less than 25, Different Periods, 1980-2004; E) Fraction of Black Mothers Aged Between 25 and 35, Different Periods, 1980-2004; F) Fraction of Black Mothers Older than 35, Different Periods, 1980-2004. Figures report point estimates of $b * 100$ in equation 1 in the main text and its associated t-statistics for different periods for several infant mortality outcomes. The first data point is for years 1980-1989, the second is for years 1981-1990, and so on until 1995-2004.

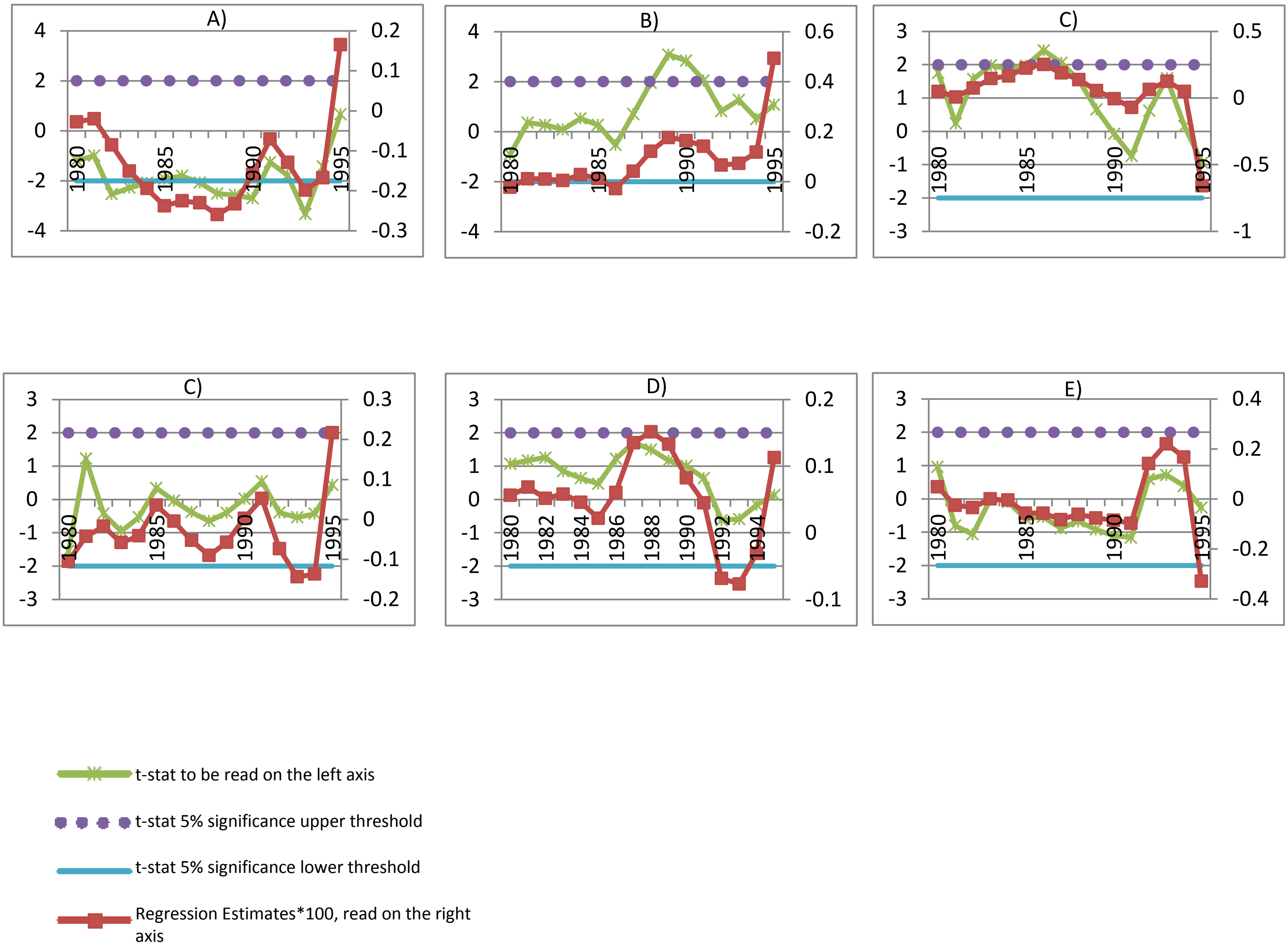

Figure K. A) Fraction of White Mothers with level of education lower than high school, Different Periods, 1980-2004; B) Fraction of White Mothers with level of education equal to high school, Different Periods, 1980-2004; C) Fraction of White Mothers with level of education higher than high school, Different Periods, 1980-2004; D) Fraction of Black Mothers with level of education lower than high school, Different Periods, 1980-2004; E) Fraction of Black Mothers with level of education higher than high school, Different Periods, 1980-2004; F) Fraction of Black Mothers with level of education higher than high school, Different Periods, 1980-2004. Figures report point estimates of $b * 100$ in equation 1 in the main text and its associated t-statistics for different periods for several infant mortality outcomes. The first data point is for years 1980-1989, the second is for years 1981-1990, and so on until 1995-2004.

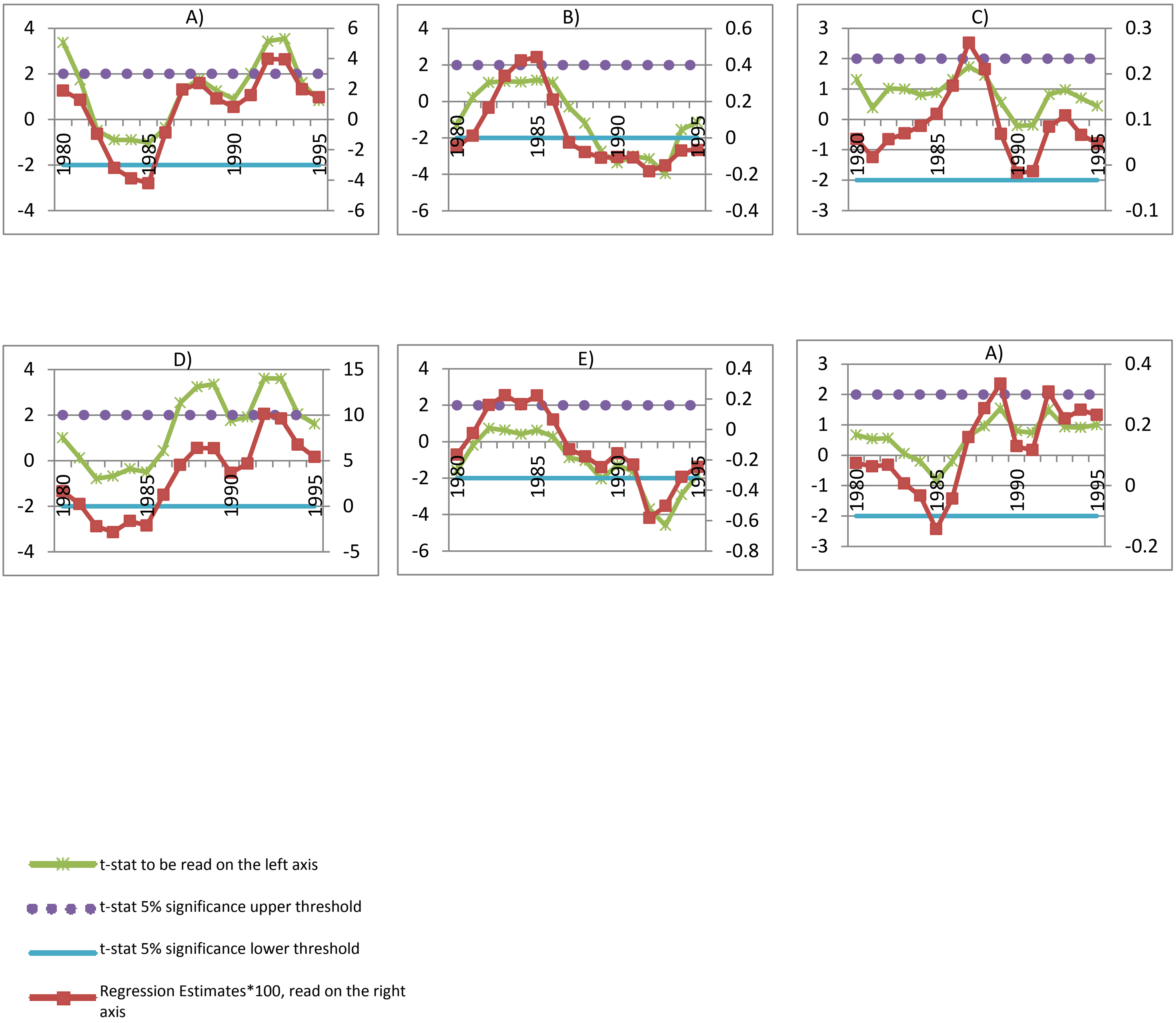

Figure L. A) Average Number of Prenatal Care visits, White Mothers, Different Periods, 1980-2004; B) Fraction of White Mothers With Less than 5 Prenatal Care Visits, Different Periods, 1980-2004; C) Fraction of White Mothers With Prenatal Care in the First Trimester, Different Periods, 1980-2004; D) Average Number of Prenatal Care visits, Black Mothers, Different Periods, 1980-2004; E) Fraction of Black Mothers With less than 5 Prenatal Care Visits, Different Periods, 1980-2004; F) Fraction of Black Mothers With Prenatal Care in the First Trimester, Different Periods, 1980-2004. Figures report point estimates of $b * 100$ in equation 1 in the main text and its associated t-statistics for different periods for several infant mortality outcomes. The first data point is for years 1980-1989, the second is for years 1981-1990, and so on until 1995-2004.

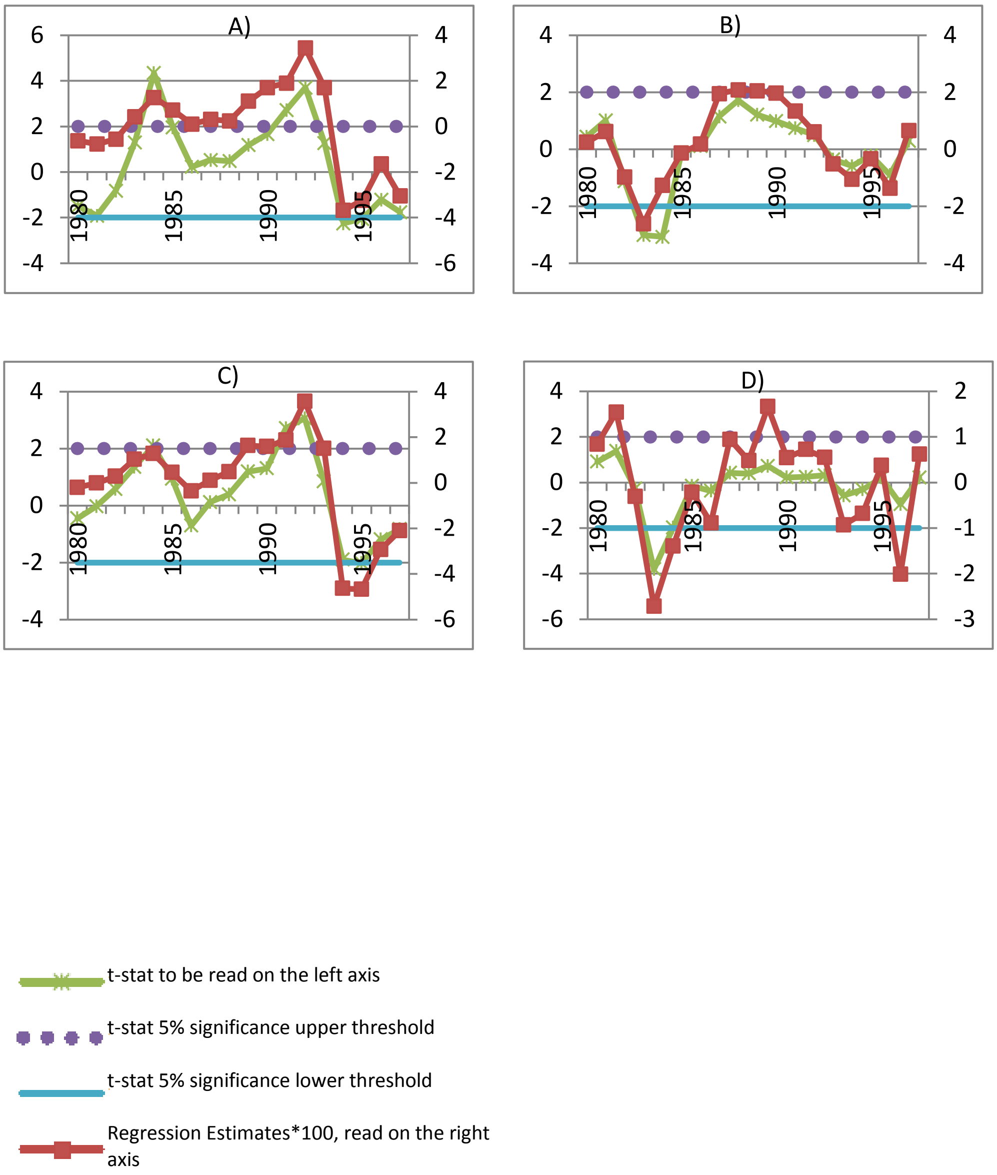

Figure M. A) White Infant Mortality, Different Periods, 1980-2004; B) Black Infant Mortality, Different Periods, 1980-2004. C) White Neonatal Mortality, Different Periods, 1980-2004. D) Black Neonatal Mortality, Different Periods, 1980-2004. Figures report point estimates of $b * 100$ in equation 1 in the main text and its associated t-statistics for different periods for several infant mortality outcomes. The first data point is for years 1980-1987, the second is for years 1981-1988, and so on until 1997-2004.
